# m6ASNP: a tool for annotating genetic variants by m^6^A function

**DOI:** 10.1093/gigascience/giy035

**Published:** 2018-04-02

**Authors:** Shuai Jiang, Yubin Xie, Zhihao He, Ya Zhang, Yuli Zhao, Li Chen, Yueyuan Zheng, Yanyan Miao, Zhixiang Zuo, Jian Ren

**Affiliations:** 1 Sun Yat-sen University Cancer Center, State Key Laboratory of Oncology in South China, Collaborative Innovation Center for Cancer Medicine, Sun Yat-sen University, Guangzhou 510060, China; 2 State Key Laboratory of Biocontrol, School of Life Sciences, Sun Yat-sen University, Guangzhou, Guangdong 510275, China; 3 Collaborative Innovation Center of High Performance Computing, National University of Defense Technology, Changsha 410073, China

**Keywords:** N6-methyladenosine (m^6^A), variant annotation, variant effect prediction, random forest

## Abstract

**Background:**

Large-scale genome sequencing projects have identified many genetic variants for diverse diseases. A major goal of these projects is to characterize these genetic variants to provide insight into their function and roles in diseases. N6-methyladenosine (m^6^A) is one of the most abundant RNA modifications in eukaryotes. Recent studies have revealed that aberrant m^6^A modifications are involved in many diseases.

**Findings:**

In this study, we present a user-friendly web server called “m6ASNP” that is dedicated to the identification of genetic variants that target m^6^A modification sites. A random forest model was implemented in m6ASNP to predict whether the methylation status of an m^6^A site is altered by the variants that surround the site. In m6ASNP, genetic variants in a standard variant call format (VCF) are accepted as the input data, and the output includes an interactive table that contains the genetic variants annotated by m^6^A function. In addition, statistical diagrams and a genome browser are provided to visualize the characteristics and to annotate the genetic variants.

**Conclusions:**

We believe that m6ASNP is a very convenient tool that can be used to boost further functional studies investigating genetic variants. The web server “m6ASNP” is implemented in JAVA and PHP and is freely available at [60].

## Introduction

Due to rapid improvements in high-throughput sequencing technology, the cost and time requirements of these technologies have been greatly reduced, which has triggered the explosive growth of high-throughput sequencing data associated with various diseases. The major goal of these high-throughput sequencing studies is to identify disease-causing variants. However, distinguishing the few disease-causing variants from the majority of passenger variants remains a major challenge. Computational methods that accurately interpret and prioritize the large amount of variants are urgently needed.

Many types of variants have different effects on the function of genes. Nonsynonymous variants, which alter the amino acids in a protein sequence, are among the most studied classes of variants. Alterations in the protein sequence can cause protein dysfunction due to a variety of different mechanisms. For example, variants in critical sites of the catalytic domain may affect protein catalytic functions [1] and variants in amino acids critical to the protein structure may affect protein-protein interactions [2], protein stability [3], and other important features [4]. Moreover, certain amino acid changes can affect post-translational modification, such as phosphorylation [5, 6], lysine modification [7], and glycosylation [8]. Currently, most bioinformatics tools mainly focus on interpreting nonsynonymous variants. For example, SIFT [9] and PolyPhen-2 [10] can predict the tolerance of nonsynonymous variants through sequence conservation; several tools, such as PhosphoSNP [11] and MIMP [12], predict whether amino acid changes affect post-translational modifications.

Compared to nonsynonymous variants, synonymous variants are neglected by most studies investigating diseases, particularly studies investigating tumors [13]. These variants are understudied because they do not alter the amino acid sequence of a protein and are considered "silent" variants. These variants are treated as "neutral" variants in evolutionary studies. However, growing evidence suggests that synonymous variants also affect the function of genes and cause various diseases [14]. Synonymous variants can result in abnormal post-transcriptional regulation, such as mRNA splicing [15], stability [16], and translation speed [17]. Many studies have shown that abnormalities in post-transcriptional regulation are closely related to genetic diseases and complex diseases [18-20]. Several bioinformatics tools that predict the effect of variants on post-transcriptional regulation are available, such as MutPred Splice [21] and SILVA [22], which primarily focus on mRNA splicing.

The post-transcriptional modification of mRNA is also an important post-transcriptional regulatory mechanism, and N6-methyladenosine (m^6^A) modification is among the most highly abundant in post-transcriptional modification [23], which regulates the metabolic processes of most RNA, including the splicing [24], stability [25], and translation of mRNA [26]. m^6^A modification is closely related to multiple diseases. Recently, FTO, an m^6^A demethylase, has been found to play an important role in the development of recessive lethality syndrome [27]. Abnormal m^6^A regulation can lead to individual developmental retardation [28], head malformations [27], mental retardation [29], brain dysfunction [30], and cardiac malformations [31]. More recently, increasing evidence has shown that dysregulation of m^6^A modification was closely related to cancer development. It was shown that abnormal modification of m^6^A and its regulators can lead to leukemia [32], prostate cancer [33], breast cancer [34, 35], bladder cancer [36], and liver cancer [37]. Therefore, it is important to evaluate the effect of variants on m^6^A modification, providing new perspective for understanding the variants, particularly for synonymous variants, thus helping to find more disease-causing variants.

A number of bioinformatics tools have been developed for predicting m^6^A sites, most of which are based on sequence characteristics. IRNA-methyl [38] and pRNAm-PC [39] used a support vector machine to construct a prediction model based on the distribution sequence characteristics. SRAMP [40] is a random forest-based tool trained on the single-nucleotide resolution m^6^A sites from miCLIP-Seq experiments [41, 42]. However, these tools are not specifically designed to deal with the variant data to evaluate the effects of the variants on m^6^A modification. It is highly desirable to develop a specific tool for predicting the effects of variant on m^6^A modification.

Here, we developed an accurate m^6^A site prediction tool that is superior to other similar tools. Based on the m^6^A site prediction tool, we constructed a web server called “m6ASNP” that is dedicated to predicting if methylation status of an m^6^A site is altered by variants around the site. We then applied m^6^ASNP to the variants collected from dbSNP.

### Data collection

To construct the prediction model, we first obtained the single-base-resolution m^6^A sites from two recently published miCLIP experiments. We collected 16,079 human m^6^A sites from Linder et al. [41] and 43,155 human m^6^A sites from Ke et al. [42]. Specifically, in Ke's paper, two tissue samples from mouse are also tested, from which we collected 8,748 and 30,078 N6-methyladenosines in liver and brain, respectively. We then combined these datasets to obtain a nonredundant dataset that contains 55,548 sites in human and 36,192 sites in mouse. For the human model, we used 35,871 nonredundant m^6^A sites as the positive training set, and the remaining 19,677 m^6^A sites were used as the positive test set. Similarly, for the mouse model, 25,334 m^6^A sites were preserved as the positive training set, and another 10,858 m^6^A sites were used as the positive test set. The negative datasets were generated according to the distribution of the positive sets. Because the majority of m^6^A sites conformed to a DRACH motif, we first defined the potential m^6^A sites as adenine sites that conform to the AC motif. Using the positive datasets as references, we extracted the nonmethylated adenines that were followed by a cytosine in the same exon as the negative dataset. From the human genome, we extracted 1904,016 adenine sites as the negative training set, while the negative test set consisted of 1,286,588 adenine sites. In the case of the mouse genome, 1,519,570 adenine sites were extracted as the negative training set and 625,600 adenine sites were constructed as the negative test set (Supplementary Data).

To decipher the potential applications of m6ASNP, we further collected a complete set of genetic variants from dbSNP for human and mouse. The single-nucleotide variations (SNVs) within the exonic regions were preserved for subsequent analysis. A total of 13,079,416 and 2,668,046 SNVs were collected in human and mouse, respectively. To investigate the potential role of these SNVs in reshaping the m^6^A event, m^6^A sites from two miCLIP-seq studies [41, 42], two PA-m^6^A-seq experiments [43], and 244 MeRIP-seq samples were integrated. Using m6ASNP, we also predicted the potential m^6^A-associated variants from the above dataset. In addition, a transcriptome-wide prediction was also performed. Overall, 311,706 and 40,308 m^6^A-associated variants were obtained from human and mouse, respectively.

In order to identify the potential roles of m^6^A-associated variants in post-transcriptome regulation, the RNA-binding protein (RBP) binding sites from starBase2 [44] and CLIPdb [45], the miRNA–RNA interactions from starBase2, and the canonical splice sites (GT-AG) from Ensembl annotations were collected. In addition, we also obtained a large number of disease-associated single-nucleotide polymorphism (SNPs) from different datasets (GWAS catalog [46], Johnson and O'Donnel [47], dbGAP [48], GAD [49], and ClinVar [50]) to perform disease-association analysis.

## Results

### Construction of m6ASNP

**Figure 1:**
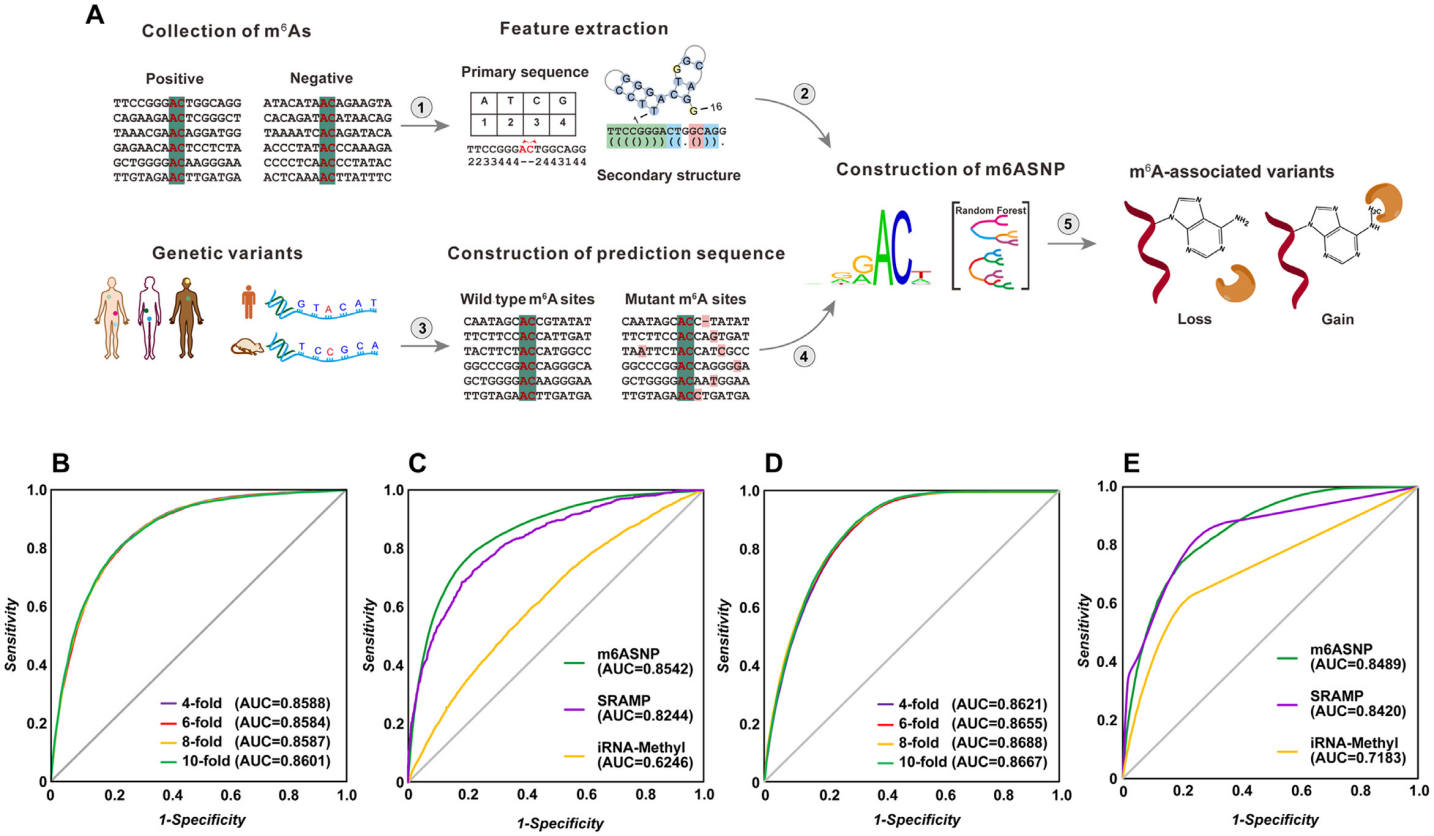
The construction of m6ASNP. A) The computational pipeline for identifying m^6^A-associated variants. (1) The single-nucleotide-resolution data were collected from recently published miCLIP-seq experiments. (2) The primary sequence and secondary structure features were extracted for subsequent model training process. (3) Genetic variants, such as somatic variants or germline SNPs, were input into the computation pipeline. (4) The flanking sequence around the potential m^6^A residue was constructed for both wild-type and mutant samples based on the inputted variants. (5) The loss and gain variants were predicted according to the above data. B) On the human model, 4-, 6-, 8-, and 10-fold cross-validation was performed. C) The performance comparison was made between m6ASNP and other state-of-the-art tools on the human test set. D) The evaluation results of 4-, 6-, 8-, and 10-fold cross-validation in mouse model. E) The performance comparison between m6ASNP and other state-of-the-art tools on the mouse test set.

As illustrated in Fig. 1A, m6ASNP was developed using a random forest algorithm (see Methods section for more details). In order to evaluate the contribution of different encoding features, we first computed the mean decrease of Gini impurity (also known as Gini importance) for the human and mouse model. The distribution plot of Gini importance in different features showed that the primary sequence was the most effective feature for predicting potential m^6^A sites. Nucleotides in the DRACH motif around the N6-methyladenosine were dominated for classification (Supplementary Fig. S1A). However, secondary structures were still observed to contribute the prediction of m^6^A sites. Further evaluation on the prediction capability of primary sequence and secondary structure indicated that the addition of structural features to the sequence features can improve the accuracy and robustness of both models (Supplementary Fig. S1B). Therefore, in the final model of both human and mouse, we combined those features to obtain a better performance. Next, to evaluate the performance of m6ASNP, 4-, 6-, 8-,and 10-fold cross-validations were performed on both the human and mouse models. In both species, the area under the curves of all the validations were close and larger than 0.84 (Fig. 1B and D), indicating that m6ASNP is an accurate and robust predictor. To further assess the prediction capability in unknown data, we then compared m6ASNP with the two other publicly available predictors, iRNA-Methyl and SRAMP, in the independent test set. As a result, the performance of m6ASNP was found to be superior to all other predictors in both the human and mouse models (Fig. 1C and E).

To balance the prediction accuracy, we selected three thresholds with high, medium, and low stringencies for classification based on the evaluation result from 10-fold cross-validation. The high, medium, and low thresholds were selected by controlling the false-positive rate at 0.05, 0.1, and 0.15, respectively. Table 1 presents the detailed performance under these three selected thresholds. In general, the high threshold provides the most stringent criterion and is usually used in large-scale prediction. The medium threshold is a balanced criterion and may be appropriate for most cases. The low threshold is the loosest criterion. When users expect to retain as many potential sites as possible, this threshold would be the best option.

**Table 1:** Prediction performance from 10-fold cross-validation under high, medium, and low thresholds

	Human					Mouse				
Threshold	*Ac*	*Sn*	*Sp*	*MCC*	*Pr*	*Ac*	*Sn*	*Sp*	*MCC*	*Pr*
**High**	0.7235	0.2781	0.9461	0.3158	0.7208	0.7154	0.2477	0.9492	0.2894	0.7092
**Medium**	0.7487	0.4497	0.8981	0.3973	0.6882	0.7465	0.4467	0.8964	0.3918	0.6832
**Low**	0.7589	0.5837	0.8465	0.4439	0.6554	0.7591	0.5956	0.8409	0.4471	0.6518

### Usage of m6ASNP

**Figure 2:**
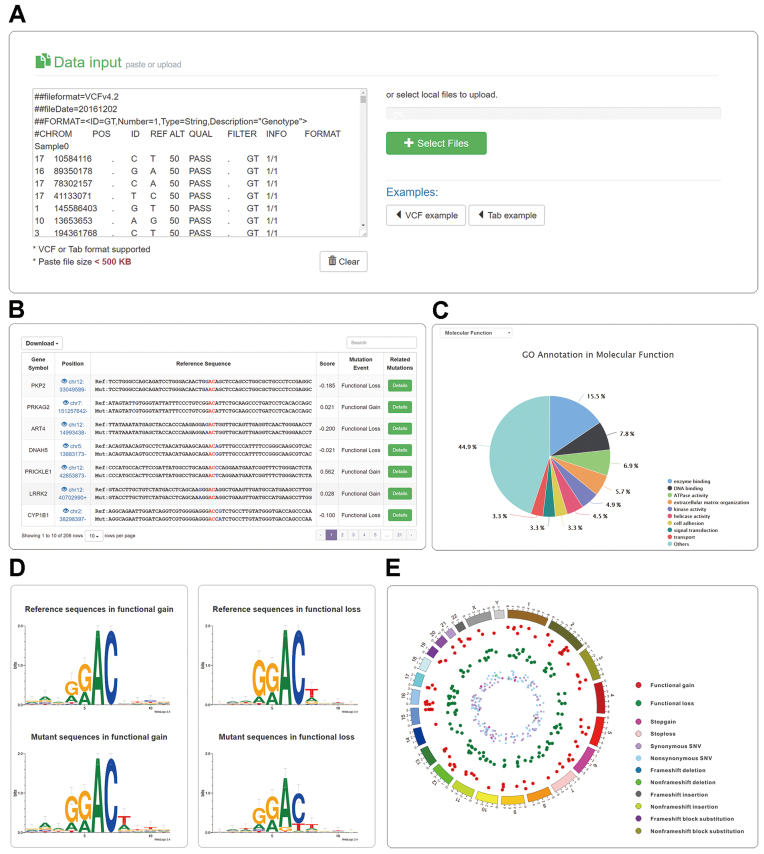
A snapshot of the m6ASNP web server. A) The main interface. Variants can be input as standard VCF format or tab-delimited flat format. A file uploading module was implemented to support large-scale prediction of m^6^A-associated variants. B) The prediction results were listed in the interactive table, allowing fast retrieval of the result data. C) The gene ontology annotation was performed on the predicted m^6^A-associated variants. D) To present the alterations of the m^6^A motif, the sequence logos were generated automatically for both functional gain and loss variants. E) The gain and loss m^6^A-associated variants, as well as the original SNPs, were illustrated in the circos plot at a genomic level by the BioCircos [51] library.

In m6ASNP, a standard variant call format (VCF) or a simplified tab delimited file are supported as input data (Fig. 2A). As an example, we applied m6ASNP to the “common and clinical” variants VCF file obtained from ClinVar that contains 7,397 variants. The predicted m^6^A-associated variants are presented in an interactive table (Fig. 2B). Of the 7,397 variants, 206 are predicted to affect the m^6^A modification, either functional gain or loss of modification. The web server will conduct a comprehensive annotation and statistical analysis for all the predicted m^6^A-associated variants. The m^6^A-associated variants from ClinVar are mainly enriched in enzyme-binding and DNA-binding gene ontology (GO) molecular functions (Fig. 2C). The sequence logos are presented to show the changes of gained and lossed m^6^A sites between the reference and mutant sequences (Fig. 2D). The “GGACU” motif is more obvious in mutant sequences compared to reference sequences for functional gain variants. While for functional loss variants, the “GGACU” motif is less noticeable in mutant sequences. A circos plot is presented to have an overview of all the m^6^A-associated variants (Fig. 2E).

### Characteristics of m^6^A-associated variants predicted by m6ASNP

**Figure 3:**
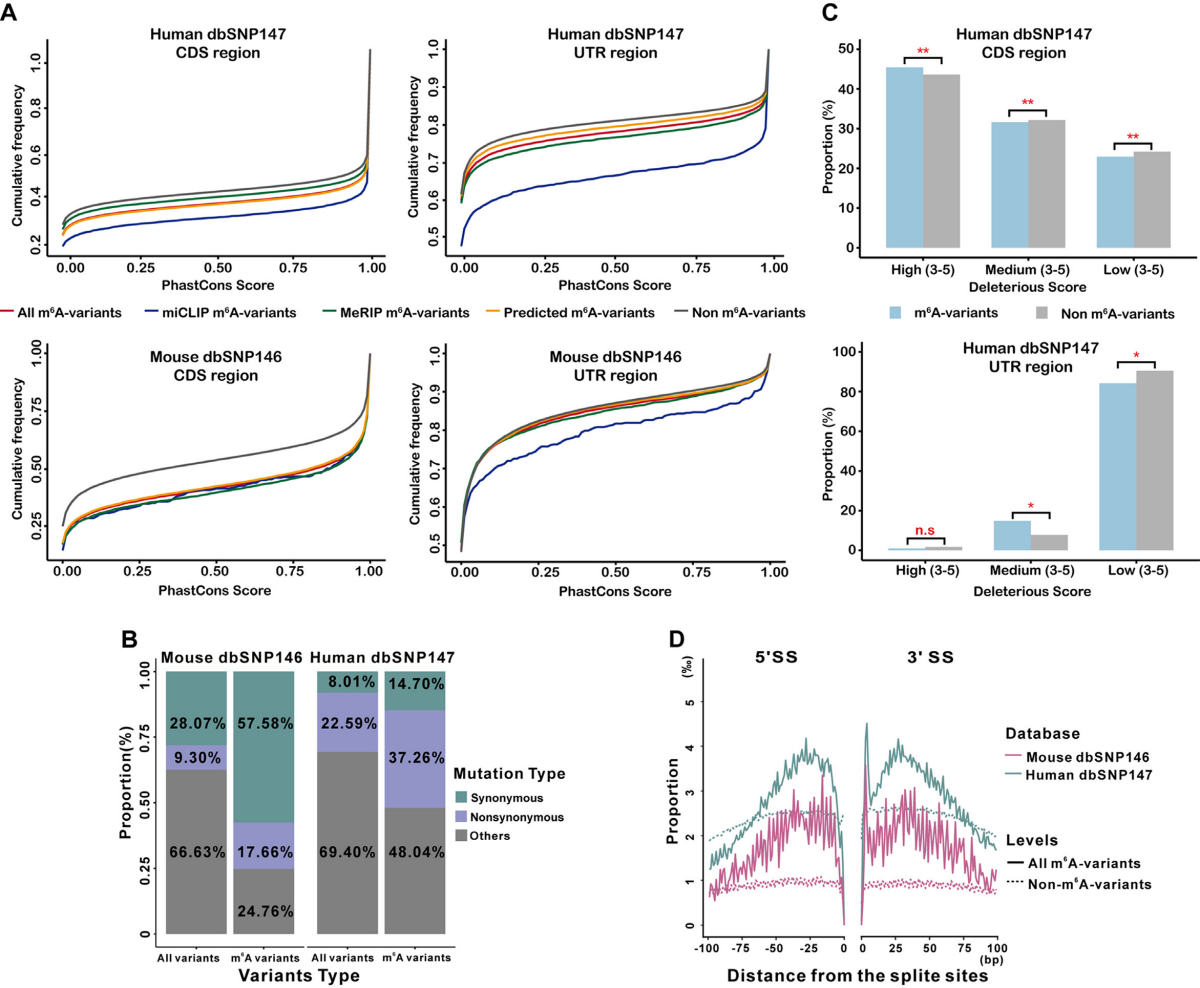
Characteristics of m^6^A-associated variants predicted by m6ASNP. A) The cumulative distribution function of phastCons score for different levels of m^6^A-associated variants and non-m^6^A variants in mouse dbSNP and human dbSNP. B) Proportional distribution of different variant types for the conserved m^6^A-associated variants. C) Proportional distribution of the m^6^A-associated variants and non-m^6^A variants at three deleterious levels predicted by a combination of five variant function predictors. A 2-tailed test of the population proportion was used to assess significance. D) Proportional distribution of m^6^A-associated variants and non-m^6^A variants at different distances from the splicing sites.

We further applied m6ASNP to all the variants in dbSNP. As a result, we obtained 133,394 functional gain and 214,884 functional loss m^6^A-associated variants. Among these m^6^A-associated variants, 6,235 located at or near the m^6^A sites from miCLIP experiments and 55,381 located at or near the m^6^A sites from MeRIP-Seq experiments. To characterize m^6^A-associated variants predicted by m6ASNP, we performed a systematic comparison between m^6^A-associated variants and non-m^6^A-associated variants (non-m^6^A variants). We found that m^6^A-associated variants were enriched in protein-coding genes (dbSNP147, 95.77%; dbSNP146, 92.12%) and significantly concentrated in CDS and 3′UTR (Supplementary Fig. S2A and Table S1). Interestingly, in both CDS and UTR regions, m^6^A-associated variants were more conserved than non-m^6^A variants (Fig. 3A). For those conserved m^6^A-associated variants, a significant portion was synonymous compared to all conserved variants (Fig. 3B, *P***<** 0.0001, hypergeometric test). To further explain the functional role of m^6^A-associated variants, we divided the predicted m^6^A-associated variants into two groups: the functional gain and functional loss variants. The conservation analysis was performed on these two groups, and the results were compared to non-m^6^A variants in both CDS and UTR regions (Supplementary Fig. S3A). Strikingly, in most cases, the functional loss variants were found to be more conservative compared to the gain variants, suggesting that the loss of existing m^6^A sites may undergo stronger selective pressure than the gain mutations on potential adenylate sites. Moreover, m^6^A-associated variants were predicted to be more deleterious than non-m^6^A variants in both the CDS and UTR regions (Fig. 3C, 2-tailed population test). Again, for the predicted data, the functional loss variants appeared to have a higher deleteriousness compared to the functional gain variants and the non-m^6^A variants (Supplementary Fig. S3B). Taken together, we conclude that m^6^A-associated variants, especially the functional loss variants, may have important roles and could be driven by positive selection in mammalian genomes. Furthermore, there were more m^6^A-associated variants located near the splice sites relative to the non-m^6^A variants, mostly distributed in the 20–30 bpflanking region of the splicing sites, implying that the variants were likely to affect RNA splicing as the means of changing the m^6^A levels (Fig. 3D). Moreover, the m^6^A-associated variants preferentially locate in genes with multiple transcripts (Supplementary Fig. S2B). These results were in agreement with the findings reported by Xiao et al. [24].

### m^6^A-associated variants in disease

Genome=wide association studies (GWAS) have revealed many disease-related variants. However, the pathogenesis mechanisms for most of these disease-related variants had not been known. We found 1,919 m^6^A-associated variants from human dbSNP were recorded either in GWAS studies or the ClinVar database. These 1,919 m^6^A-associated variants were related to various diseases, including cardiovascular phenotype, muscular dystrophy, tuberous sclerosis syndrome, and cancer. Of them, hereditary cancer (436 variants, 22.74%, *P* = 2.27e-30, Chi-squared test), Familial breast cancer (96 variants, 5.01%; *P* = 8.33e-9, Chi-squared test) and hereditary nonpolyposis colorectal cancer (73 variants, 3.81%; *P* = 5.5e-5, Chi-squared test) were the top enriched disease types (Supplementary Table S2). Our findings provide insights into the potential pathogenesis mechanism for many disease-related variants whose functions were not clear before.

Synonymous variants have been neglected in most previous studies of disease. Since m6ASNP can be used to predict the effect of both nonsynonymous and synonymous variants, this tool could significantly supplement the function of current annotating tools that mainly focus on nonsynonymous variants. Indeed, among the m^6^A-associated variants predicted by m6ASNP, 59.86% and 25.67% are synonymous variants in mouse dbSNP and human dbSNP, respectively. By using m6ASNP, we identified many m^6^A-associated synonymous variants that have been shown to be disease related. For instance, rs139362268, a synonymous variant of *PALB2*, is related to breast cancer and pancreatic cancer. Interestingly, we observed that rs139362268 occurred in the m^6^A site of *PALB2*, in which m^6^A peaks were detected in six MeRIP-Seq experiments (Supplementary Fig. S4A). We speculated that the cancer-related synonymous variant rs139362268 might be functional through dysregulation of m^6^A modification.

### m^6^A-associated variants in post-transcriptional regulation

It has been reported that m^6^A sites could recruit RBPs that play critical roles in post-transcriptional regulations [52]. We systematically examined the genomic positional relationship between m^6^A-associated variants and RBPs to determine whether m^6^A-associated variants function through RBPs. We found the m^6^A-associated variants were significantly enriched in RBP-binding regions compared to the non-m^6^A variants (Supplementary Fig. S4B). More than 50% of the human m^6^A-associated variants were located within RBP-binding regions. We found 19 RBPs were significantly overlapped with the regions having m^6^A-associated variants (Supplementary Table S3). As expected, the m^6^A reader YTHDF2 and m^6^A eraser ALKBH5 were significantly overlapped with the regions having m^6^A-associated variants compared to the randomly selected regions. Moreover, GO annotations demonstrated that these RBPs are enriched in RNA splicing, RNA translation, and miRNA regulation (Supplementary Table S3). Among them, SFRS1, a known splicing factor, is reportedly involved in alternative splicing and is co-localized with ALKBH5 in a demethylation-dependent manner, suggesting it might participate in the regulation of RNA methylation [53].

It has been reported that m^6^A sites are enriched in miRNA target sites and regulated by miRNAs [54]. Consistent with this, we found m^6^A-associated variants predicted by m6ASNP occurred significantly more frequently in miRNA target sites than the non-m^6^A variants (Supplementary Fig. S4C). The miRNAs with a significant number of m^6^A-associated variants are listed in Supplementary Table S4. Among them, *miR-132-3p* and *miR-212-3p* were mainly expressed in the brain and played critical roles in neuronal functions as well as circadian clock entrainment [55], which is consistent with m^6^A function [56]. Interestingly, m^6^A-associated variants related to *miR-132-3p* and *miR-212-3p* were identified in both human and mouse, suggesting a conservation of function in these variants.

## Discussion

There is growing evidence that aberrant m^6^A modification is a potential pathogenesis mechanism in many diseases including cancer, which suggests the variants that disrupt m^6^A modification might cause diseases. However, currently there is still a lack of methodology for annotating variants from high-throughput sequencing studies by m^6^A function. To address this, we developed a novel computation model, m6ASNP, that is dedicated to predicting the variants that disrupt m^6^A modification. Using m6ASNP, we performed further functional analysis on m^6^A-associated variants. By integrating dataset regarding RBP-binding regions, miRNA-targets and splicing sites, m6ASNP can help to reveal the potential relationship among variants, m^6^A modification, and other post-transcriptional regulation. Also, in the disease-association analysis, more than 2,000 disease-related variants that may be linked with alterations of m^6^A modification were identified. This finding further proves that m6ASNP is a promising tool for studying the potential role of m^6^A variants in clinical investigation.

In conclusion, m6ASNP is a useful computational web server for annotating variants by m^6^A function. m6ASNP will serve as a supplemental method to run in parallel with other annotating tools to comprehensively predict the function of the variants, for both synonymous and nonsynonymous, in the high-throughput sequencing studies of diseases.

## Methods

### Construction of m^6^A site prediction model

The sequences of the flanking regions 30 nucleotides upstream and downstream of a given m^6^A residue were extracted. To transform the primary sequences to numeric vectors, each nucleotide was encoded by four distinct variables. In total, 60 numeric variables were generated for a single m^6^A residue. As reported in recent studies [57, 58], specific RNA secondary structures around the potential adenosines can affect the enzymatic process of RNA methylation. We therefore added secondary structure features to our prediction model. Using the Nussinov algorithm [59], we first predicted the secondary structure for each m^6^A residue and marked the structure state (paired or not paired) with a bracket or dot. For example, a given m^6^A nucleotide with the sequence TTCCGGGACTGGCAGG could be represented as (((())))((.())). Next, we extracted the secondary structure triplet, formed by the structure state of the three adjacent nucleotides obtained from the predicted RNA structure. The number of occurrences of each triplet in the sequence was counted and normalized to produce a 27-dimension feature vector. Combining all the primary sequences and secondary structure features, we constructed an 87-dimension vector for each m^6^A residue. These vectors were subsequently used as the input for a random forest classifier for training and prediction.

The random forest classifier for human and mouse were trained separately on the above-collected training set. The tree number was optimized as 500 and the features used for each splitting were set to 9. To assess the performance, we used 4-, 6-, 8-, and 10-fold cross-validation on the training set. The additional test set was also applied in our study to evaluate the robustness. The sensitivity, specificity, and Matthew's correlation coefficient were used to measure the predictor's performance.

### Construction of m^6^ASNP

Based on the m^6^A site prediction model, we then developed a computational pipeline to predict the effect of variants on m^6^A modification. First, variants were mapped to known transcripts. The wild-type and mutant form of the transcript sequences were then generated for m^6^A site prediction. For an m^6^A site that occurred in the wild-type transcript and disrupted in the mutant transcript, we defined it as an m^6^A-associated loss variant. The m^6^A-associated gain variant was conversely formed. To measure the altered degree of m^6^A modifications, equation 1 was defined as follows:

(1) \begin{equation*}
S = ln\left( {\frac{{RF\_Score}_{wild{-}type}}{{RF\_Score}_{mutant}}} \right)
\end{equation*}

where S denotes the alteration score that quantitatively represented the degree of m^6^A alterations between reference and mutant samples and *RF_Score* is the predicted score of a given m^6^A site from the random forest model. Obviously, alteration scores higher than 0 represent m^6^A-gain alterations, while scores lower than 0 represent m^6^A-loss alterations. In some m^6^A-associated loss variants, alteration scores were assigned to MAX, which means that the core AC motif is destroyed by genetic variants, leading to complete losses of m^6^A at those sites.

To provide convenience to the research community, we developed a web server called “m6ASNP” to specifically predict the effect of variants on m^6^A modification. m6ASNP was implemented using JAVA and PHP and is freely accessible at [60].

### Derivation of the m^6^A-associated variants

Based on miCLIP-seq, PA-m^6^A-seq, and MeRIP-seq data, we then combined them with the SNV data from dbSNP and performed m^6^A-association prediction using m6ASNP. Following the same procedure proposed in our previously published work [61], we constructed three confidence levels of annotations of m^6^A-associated variants for subsequent analysis.

The first annotation was the high-confidence-level data that contained the m^6^A-associated variants derived from miCLIP-seq and PA-m^6^A-seq experiments. Notably, the PA-m^6^A-seq can only detect m^6^A signal in a resolution of ∼23 nt. Therefore, in order to obtain precise modification sites, we scanned through all the peak regions and extracted adenosine sites that conformed to DRACH motif as the final m^6^A sites. On this basis, we retained the variants that located near the m^6^A sites as the m^6^A-associated variants.

The second annotation was the medium-confidence-level data. We first downloaded all the published MeRIP-seq data from the GEO database. According to the standard analysis pipeline for MeRIP-seq data, we applied MACS2 [62], MeTPeak [63], and Meyer's method [64] to identify the m^6^A peaks in each study separately. In general, in MeRIP-seq experiments, if a given region is identified as enriched in most of the adopted methods, it is more likely to be a true modification signal. Therefore, to obtain reliable m^6^A peaks, a tool called MSPC [65] was then applied to construct consensus peaks from the above three methods. In those consensus peaks, we then applied m6ASNP to predict m^6^A-associated variants that significantly change the DRACH motif.

The third annotation was the low-confidence-level data, where we used the whole transcriptome sequences for prediction. With a high threshold, m6ASNP will predict the potential m^6^A-associated variants from all collected genetic variants.

In summary, we constructed 13,703 high-confidence-level, 54,222 medium-confidence-level, and 243,880 low-confidence-level m^6^A-associated variants for human. Another 935 high-confidence-level, 9,404 medium-confidence-level,and 17,739 low-confidence-level data were also constructed for mouse.

### Annotation of m^6^A-associated variants

All the identified m^6^A-associated variants were annotated by the transcript structure, including the CDS, 3′ UTR, 5′ UTR, start codon, and stop codon. For the annotation of noncoding RNA DASHR [66], miRBase (version 21) (miRBase, RRID:SCR_003152) [67], GtRNAdb [68], and piRNABank [69] were used. To test whether the m^6^A-associated variants were more preferentially distributed in specific transcript structures, we calculated the proportion of variants that located in a given transcript structure. In order to avoid bias, only the variants that were annotated in mRNA were used, and the proportion in 5′-UTR, CDS, and 3′-UTR were calculated. A 2-tailed proportion test was then adopted to compare the proportion difference between m^6^A-associated variants and non-m^6^A variants. In addition, in order to evaluate their conservation scores and deleteriousness, we further annotated the m^6^A-associated variants by ANNOVAR (updated 1 February 2016) (ANNOVAR, RRID:SCR_012821) [70]. To avoid any bias, we only preserved those variants located in mRNA for analysis and compared the conservative and deleterious differences between m^6^A-associated variants and non-m^6^A variants in the same exon. As the selective pressures were quite different in protein-coding sequences and untranslated regions, the above comparison was carried out separately for the CDS and UTR regions. Specifically, the conservation scores were calculated by phastCons with 100-way and 60-way gene conservation profiles for the human and mouse, respectively [71]. The deleteriousness of each variants was measured by integrating the prediction results from five pieces of software (SIFT [72], PolyPhen2 HVAR [10], PolyPhen2 HDIV [10], LRT [73], and FATHMM [74]). We defined an aggregate score by counting the number of above-listed methods that consider an SNV to be deleterious. A deleterious score of 0 means that the variant is predicted to be tolerated in all methods, while a deleterious score of 5 means that the corresponding variant is predicted to be deleterious in all five predictors. As a result, the aggregate score may range from 0 to 5, and a higher score indicates a higher probability of deleterious.

### Disease-association analysis

A linkage disequilibrium (LD) analysis was performed for each GWAS disease-associated SNP. We used Haploview (Haploview, RRID:SCR_003076) to obtain the LD mutations using a parameter r^2^ > 0.8 in at least one of the four populations from CHB, CEU, JPT, and TSI. Then, we selected all m^6^A-associated variants by mapping the variants to GWAS disease-associated SNPs and their LD mutations. Moreover, we collected ClinVar data to annotate the m^6^A-associated variants with specific functions.

### Post-transcriptional regulation association analysis

First, the m^6^A-associated variants were intersected with the collected RBP regions for the same sample. We matched all m^6^A-associated variants with miRNA targets to obtain the m^6^A-associated variants that potentially impacted the miRNA-target interactions. Additionally, we extracted 100 bp upstream of the 5′ splicing sites and 100 bp downstream of the 3′ splicing sites. Subsequently, we matched the m^6^A-associated variants to these regions to obtain the splicing sites affected by the m^6^A-associated variants.

### Identification of significant RBPs and miRNAs

To determine whether the m^6^A-associated variants were significantly enriched in RBP regions, an empirical evaluation was performed for each RBP. Using YTHDF2 as an example, the process may be described as follows.

First, we calculated the number of m^6^A-associated variants within the YTHDF2-binding regions (defined as *N_RBP_*). Second, because certain m^6^A-associated variants randomly occur within the YTHDF2-binding regions, we estimated the background count of m^6^A-associated variants for YTHDF2 (defined as *N_B_*). Thus, we extracted the longest transcript for each gene from the gene annotation files. The weight of the *i*th gene was defined as follows:

(2) \begin{equation*}
{{w(i) = }}\frac{{{{L(i)}}}}{{\sum\limits_{{{i = 0}}}^{{n}} {{{L(i)}}} }}
\end{equation*}

(3) \begin{equation*}
\sum\limits_{{{i = 0}}}^{{n}} {{{w(i) = 1}}}
\end{equation*}

where *n* was the total number of genes annotated and *L*(*i*) was the length (bp) of the *i*th gene. Then, we extracted the same-length reads of all YTHDF2-binding regions, which was defined as *N_B_*, using weighted random sampling of all transcripts collected above. We repeated this procedure 50,000 times and then obtained the frequency *F_RBP_* when N_B_ was greater than *N_RBP_* in the cycle. This frequency may be regarded as an estimation of the probability that observing *N_B_* greater then *N_RBP_*in random condition. Next, the Benjamini-Hochberg method was applied to control the false positives. An adjusted *F_RBP_* less than 0.05 was considered a small probability event, suggesting that the m^6^A-associated variants were more likely to occur in the RBP-binding regions of YTHDF2. All significant RBPs are listed in Supplementary Table S2. Certain significant miRNAs, which are listed in Supplementary Table S3, were obtained by performing a similar analysis of miRNA targets.

## Availability of supporting source code and requirements

Project name: m6ASNP

Project home page: https://m6asnp.renlab.org

https://github.com/RenLabBioinformatics/m6ASNP

RRID:SCR_016048

Operating system(s): platform independent

Programing language: PHP, java, javascript

License: GPLv3

## Availability of supporting data

The training data and test data collected from Linder *et al*. and Ke *et al*. are available in the supplementary data. These and snapshots of the code are also available in the *GigaScience* GigaDB repository [75].

## Additional files

**Supplementary Figure S1:** The feature contribution of the human and mouse model. Distribution plot of the feature's Gini importance for both (A) human and (B) mouse model. The prediction capabilities of different combination of features for (C) human and (D) mouse model.

**Supplementary Figure S2:** A systematic comparison of the m^6^A-associated variants and non-m^6^A variants. (A) Proportional distribution of the variants at different m^6^A confidence levels and non-m^6^A variants located in the CDS and 3′ UTR. A two-tailed test of population proportion was performed to assess significance. (B) Boxplots show the gene isoforms of the m^6^A-associated variants and non-m^6^A variants in different databases. One-sided Wilcoxon signed-rank test was performed to determine the significance. “**” indicates a significance level of *P* ≤ 0.01, while “*” indicates *P* ≤ 0.05.

**Supplementary Figure S3:** The characteristics of m6A-associated variants predicted by m6ASNP. (A) The conservation differences between functional gain and functional loss variants. (B) The comparison of mutation deleteriousness between functional gain and functional loss variants.

**Supplementary Figure S4:** Association analysis of m6A-associated variants. (A) An example of m^6^A-associated variants in disease. The red rectangle in exon 10 represents a synonymous mutation in *PALB2*, i.e., rs139362268, while the green rectangle represents the m^6^A site. The 1 to 6 numbering indicates the different samples, followed by HepG2, GM12878, Momo-mac-6, HeLa, shMETTL14 in A549 and shGFP in A549. MeRIP-seq peak tracks of input, and the IP samples were scaled to the same level and colored in red and blue. (B-C) Proportional distribution of different levels of m^6^A-associated variants and non-m^6^A variants located within the RBP-binding regions and miRNA target regions. A two-tailed test of population proportion was performed to assess significance. “**” indicates a significance level of *P* ≤ 0.01, while “*” indicates a significance level of *P* ≤ 0.05.

**Supplementary Table S1:** The distribution characteristics of m6A-associated variants in different transcript structures.

**Supplementary Table S2:** Significant disease phenotypes in m6A-associated.

**Supplementary Table S3:** Significant RBPs in m6A-associated variants Supplementary Table S3. Significant RBPs in m6A-associated variants.

**Supplementary Table S4:** Significant miRNAs in m6A-associated variants.

**Supplementary Data 1**. Single nucleotide resolution m6A sites (Training data, hg19).

## Abbreviations

GO, Gene ontology; GWAS, Genome-wide association study; LD, Linkage disequilibrium; m^6^A, N6-methyladenosine; RBP, RNA-binding protein; SNP, single nucleotide polymorphism; VCF, Variant call format.

## Ethics approval and consent to participate

Not applicable.

## Disclosure statement

The authors declare that they have no competing interests.

## Supplementary Material

GIGA-D-17-00348_Original_Submission.pdf

GIGA-D-17-00348_Revision_1.pdf

GIGA-D-17-00348_Revision_2.pdf

GIGA-D-17-00348_Revision_3.pdf

Response_to_Reviewer_Comments_Original_Submission.pdf

Response_to_Reviewer_Comments_Revision_1.pdf

Response_to_Reviewer_Comments_Revision_2.pdf

Reviewer_1_Report_(Original_Submission) -- Yun-Gui Yang 1/3/2018 Reviewed

Reviewer_1_Report_(Revision_1) -- Yun-Gui Yang 2/11/2018 Reviewed

Reviewer_2_Report_(Original_Submission) -- Bastian Linder 18 Jan 2018 Reviewed

Reviewer_2_Report_(Revision_1) -- Bastian Linder 2/22/2018 Reviewed

Supplemental material
