# Supplementary material for: m6ASNP: a tool for annotating genetic variants by m6A function
Source: Gigascience. 2018 Apr 2;7(5):giy035. doi: 10.1093/gigascience/giy035 (PMC6007280; doi:10.1093/gigascience/giy035)
Supplement: GIGA-D-17-00348_Revision_3.pdf [file giy035_giga-d-17-00348_revision_3.pdf]

GigaScience

m6ASNP: a tool for annotating genetic variants by m6A function

--Manuscript Draft--

**Manuscript Number:**	GIGA-D-17-00348R3
**Full Title:**	m6ASNP: a tool for annotating genetic variants by m6A function
**Article Type:**	Technical Note
**Funding Information:**	National Key Research and Development Program (2017YFA0106700) — Dr. Zhixiang Zuo<br>National Natural Science Foundation of China (31771462) — Dr. Jian Ren
**Abstract:**	Background<br>Large-scale genome sequencing projects have identified many genetic variants for diverse diseases. A major goal of these projects is to characterize these genetic variants to provide insight into their function and roles in diseases. N6-methyladenosine (m6A) is one of the most abundant RNA modifications in eukaryotes. Recent studies have revealed that aberrant m6A modifications are involved in many diseases.<br>Findings<br>In this study, we present a user-friendly web server called "m6ASNP" that is dedicated to the identification of genetic variants targeting m6A modification sites. A random forest model was implemented in m6ASNP to predict whether the methylation status of an m6A site is altered by the variants surrounding the site. In m6ASNP, genetic variants in a standard VCF format are accepted as the input data, and the output includes an interactive table containing the genetic variants annotated by m6A function. In addition, statistical diagrams and a genome browser are provided to visualize the characteristics and annotate the genetic variants.<br>Conclusions<br>Altogether, we believe that m6ASNP is a highly convenient tool that can be used to boost further functional studies investigating genetic variants. The web server "m6ASNP" is implemented in JAVA and PHP and is freely available at http://m6asnp.renlab.org.<br>KEYWORDS: N6-methyladenosine (m6A), variant annotation, variant effect prediction, random forest
**Corresponding Author:**	Zhixiang Zuo<br><br>Guangzhou, Please Select CHINA
**Corresponding Author Secondary Information:**	
**Corresponding Author's Institution:**	
**Corresponding Author's Secondary Institution:**	
**First Author:**	Shuai Jiang
**First Author Secondary Information:**	
**Order of Authors:**	Shuai Jiang
	Yubin Xie
	Zhihao He
	Ya Zhang
	Yuli Zhao
	Yueyuan Zheng
	Yanyan Miao
	Zhixiang Zuo

Powered by Editorial Manager® and ProduXion Manager® from Aries Systems Corporation

	Jian Ren
**Order of Authors Secondary Information:**	
**Response to Reviewers:**	Dear Editors,<br><br>We have rearranged the orders for the supplementary files.<br><br>Best,<br><br>Zhixiang
**Additional Information:**	
**Question**	**Response**
Are you submitting this manuscript to a special series or article collection?	No
**Experimental design and statistics**<br><br>Full details of the experimental design and statistical methods used should be given in the Methods section, as detailed in our Minimum Standards Reporting Checklist. Information essential to interpreting the data presented should be made available in the figure legends.<br><br>Have you included all the information requested in your manuscript?	Yes
**Resources**<br><br>A description of all resources used, including antibodies, cell lines, animals and software tools, with enough information to allow them to be uniquely identified, should be included in the Methods section. Authors are strongly encouraged to cite Research Resource Identifiers (RRIDs) for antibodies, model organisms and tools, where possible.<br><br>Have you included the information requested as detailed in our Minimum Standards Reporting Checklist?	Yes
**Availability of data and materials**<br><br>All datasets and code on which the conclusions of the paper rely must be either included in your submission or deposited in publicly available repositories (where available and ethically appropriate), referencing such data using a unique identifier in the references and in the "Availability of Data and Materials"	Yes

section of your manuscript.<br><br>Have you have met the above requirement as detailed in our Minimum Standards Reporting Checklist?	

Manuscript

Click here to download Manuscript m6ASNP manuscript_SCE.docx

m6ASNP: a tool for annotating genetic variants by m^{6}A function

Shuai Jiang[1,2,†], Yubin Xie[2,†], Zhihao He[2,†], Ya Zhang[2], Yuli Zhao[2], Li Chen[2], Yueyuan Zheng[2], Yanyan Miao[2], Zhixiang Zuo[1,*], Jian Ren[1,2,3,*]

[1]Sun Yat-sen University Cancer Center, State Key Laboratory of Oncology in South China, Collaborative Innovation Center for Cancer Medicine, Sun Yat-sen University, Guangzhou 510060, China

[2]State Key Laboratory of Biocontrol, School of Life Sciences, Sun Yat-sen University, Guangzhou, Guangdong 510275, China

[3]Collaborative Innovation Center of High Performance Computing, National University of Defense Technology, Changsha 410073, China

Shuai Jiang: jiang22@mail2.sysu.edu.cn

Yubin Xie: xieyb3@mail2.sysu.edu.cn

Zhihao He: hezhh5@mail2.sysu.edu.cn

Ya Zhang: zhangya6@mail2.sysu.edu.cn

Yuli Zhao: zhaoyli3@mail2.sysu.edu.cn

Yueyuan Zheng: zhyuey2@mail2.sysu.edu.cn

Yanyan Miao: miaoyany@mail.sysu.edu.cn

†Contributed equally

*Correspondence to: Tel/Fax: +86 20 87342325; Jian Ren: renjian.sysu@gmail.com, ORCID: 0000-0002-4161-1292; Zhixiang Zuo: zuozhx@sysucc.org.cn, ORCID: 0000-0002-2492-2689.

Abstract

Background

Large-scale genome sequencing projects have identified many genetic variants for diverse diseases. A major goal of these projects is to characterize these genetic variants to provide insight into their function and roles in diseases. N6-methyladenosine (m^6A) is one of the most abundant RNA modifications in eukaryotes. Recent studies have revealed that aberrant m^6A modifications are involved in many diseases.

Findings

In this study, we present a user-friendly web server called "m6ASNP" that is dedicated to the identification of genetic variants targeting m^6A modification sites. A random forest model was implemented in m6ASNP to predict whether the methylation status of a m^6A site is altered by the variants surrounding the site. In m6ASNP, genetic variants in a standard VCF format are accepted as the input data, and the output includes an interactive table containing the genetic variants annotated by m^6A function. In addition, statistical diagrams and a genome browser are provided to visualize the characteristics and annotate the genetic variants.

Conclusions

We believe that m6ASNP is a highly convenient tool that can be used to boost further functional studies investigating genetic variants. The web server "m6ASNP" is implemented in JAVA and PHP and is freely available at http://m6asnp.renlab.org.

KEYWORDS: N6-methyladenosine (m^6A), variant annotation, variant effect prediction, random forest

Introduction

Due rapid improvements in high-throughput sequencing technology, the cost and time requirements of these technologies have been greatly reduced, which has triggered the explosive growth of high-throughput sequencing data associated with various diseases. The major goal of these high-throughput sequencing studies is to identify disease-causing variants. However, distinguishing the few disease-causing variants from the majority of passenger variants remains a major challenge. Computational methods that accurately interpret and prioritize the large amount of variants are urgently needed.

Many types of variants have different effects on the function of genes. Non-synonymous variants, which alter the amino acids in a protein sequence, are among the most studied classes of variants. Alterations in the protein sequence can cause protein dysfunction due to a variety of different mechanisms. For example, variants in critical sites of the catalytic domain may affect protein catalytic functions [1]; variants in amino acids critical to the protein structure may affect protein-protein interactions [2], protein stability [3] and other important features [4]. Moreover, certain amino acids changes can affect post-translational modification, such as phosphorylation [5, 6], lysine modification [7] and glycosylation [8]. Currently, most bioinformatics tools mainly focus on interpreting non-synonymous variants. For example, SIFT [9] and PolyPhen-2 [10] can predict the tolerance of non-synonymous variants through sequence conservation; several tools, such as PhosphoSNP [11] and MIMP [12], predict whether amino acids changes affect post-translational modifications.

Compared to non-synonymous variants, synonymous variants are neglected by most studies investigating diseases, particularly studies investigating tumors [13]. These variants are understudied because they do not alter the amino acid sequence of a protein and are considered "silent" variants. These variants are treated as "neutral" variants in evolutionary studies. However, growing evidence suggests that synonymous variants also affect the function of genes and cause various diseases [14]. Synonymous variants can result in abnormal post-transcriptional regulation, such as mRNA splicing [15], stability [16] and translation speed [17]. Many studies have shown that abnormalities in post-transcriptional regulation are closely related to genetic diseases and complex diseases [18-20]. Several bioinformatics tools that predict the effect of variants on post-transcriptional regulation are available, such as MutPred Splice [21] and SILVA [22], which primarily focus on mRNA splicing.

The post-transcriptional modification of mRNA is also an important post-transcriptional regulatory mechanism, and N6-methyladenosine (m^6A) modification is among the most highest abundances in post-transcriptional modification [23], which regulates the metabolic processes of most RNA, including the splicing [24], stability [25] and translation of mRNA [26]. m^6A modification is closely related to multiple diseases. Recently, FTO, an m^6A demethylase, have been found to play an important role in the development of recessive lethality syndrome [27]. Abnormal m^6A regulation can lead to individual developmental retardation [28], head malformations [27], mental retardation [29], brain dysfunction [30] and cardiac malformations [31]. More recently, increasing evidence has shown that dysregulation of m^6A modification was closely related to cancer development. It was shown that abnormal of m^6A modification and its regulators can lead to leukemia [32], prostate cancer [33], breast cancer [34, 35], bladder cancer [36] and liver cancer [37]. Therefore, it is important to evaluate the effect of variants on m^6A modification, providing new perspective of understanding the variants, particularly for those synonymous variants, thus help finding more disease-causing variants.

There exists a number of bioinformatics tools developed for predicting m^6A sites, most of which are based on sequence characteristics. IRNA-Methyl [38] and pRNAm-PC [39] utilized support vector machine (SVM) to construct a prediction model based on the distribution sequence characteristics. SRAMP [40] is a Random Forest based tool trained on the single-nucleotide resolution m^6A sites from miCLIP-Seq experiments [41, 42]. However, these tools are not specifically designed to deal with the variant data to evaluate the effects of the variants on m^6A modification. It is highly desirable to develop a tool specifically for predicting the effects of variant on m^6A modification.

In this paper, we first developed an accurate m^6A site prediction tool that is superior to other similar tools. Based on m^6A site prediction tool, we constructed a webserver called "m6ASNP" that is dedicated to predict if methylation status of an m^6A site is altered by variants around the site. We then applied m^6ASNP to the variants collected from dbSNP.

Data collection

To construct the prediction model, we first obtained the single-base-resolution m^6A sites from two recently published miCLIP experiments. We collected 16,079 human m^6A sites from Linder *et al* [41] , and 43,155 human m^6A sites from Ke *et al* [42]. Specifically, in Ke's paper, two tissue samples from

mouse are also tested, from which we collected 8748 and 30078 N6-methyladenosines in liver and brain, respectively. We then combined these data sets to obtain a non-redundant data set that contains 55,548 sites in human and 36,192 sites in mouse. For human model, we used 35,871 non-redundant m^6A sites as positive training set, and the rest 19,677 m^6A sites were used as positive test set. Similarly, for mouse model, 25,334 m^6A sites were preserved as positive training set, and another 10,858 m^6A sites were used as positive test set. The negative data sets were generated according to the distribution of the positive sets. Because the majority of m^6A sites conformed to a DRACH motif, we first defined the potential m^6A sites as adenine sites that conform to the AC motif. Using the positive data sets as references, we extracted the non-methylated adenines that were followed by a cytosine in the same exon as the negative data set. From the human genome, we extracted 1,904,016 adenine sites as the negative training set, while the negative test set consisted of 1,286,588 adenine sites. In the case of mouse genome, 1,519,570 adenine sites were extracted as negative training set and 625,600 adenine sites were constructed as negative test set (**Supplementary Data**).

To decipher the potential applications of m6ASNP, we further collected a complete set of genetic variants from dbSNP for human and mouse. The single-nucleotide variations (SNVs) within the exonic regions were preserved for subsequent analysis. Totally, 13,079,416 and 2,668,046 SNVs were collected in human and mouse, respectively. To investigate the potential role of these SNVs in reshaping the m^6A event, m^6A sites from two miCLIP-seq studies [41, 42], two PA-m^6A-seq experiments [43] and 244 MeRIP-seq samples were integrated. Using m6ASNP, we further predicted the potential m^6A-associated variants from the above data set. Besides, a transcriptome-wide prediction was also performed. Overall, 311,706 and 40,308 m^6A-associated variants were obtained from human and mouse, respectively.

In order to identify the potential roles of m^6A-associated variants in post-transcriptome regulation, the RBP binding sites from starBase2 [44] and CLIPdb [45], the miRNA–RNA interactions from starBase2 and the canonical splice sites (GT-AG) from Ensembl annotations were collected. In addition, we also obtained a large number of disease-associated SNPs from different data sets (GWAS catalog [46], Johnson and O’Donnel [47], dbGAP [48], GAD [49] and ClinVar [50]) to perform disease-association analysis.

Results

Construction of m6ASNP

As illustrated in **Fig. 1A**, m6ASNP was developed using random forest algorithm (see methods for detail). In order to evaluate the contribution of different encoding features, we first computed the mean decrease of Gini impurity (also known as Gini importance) for the human and mouse model. The distribution plot of Gini importance in different features showed that the primary sequence was the most effective feature for predicting potential m^6A sites. Nucleotides in the DRACH motif around the N6-methyladenosine were dominated for classification (**Fig S1A**). However, secondary structures were still observed to contribute the prediction of m^6A sites. Further evaluation on the prediction capability of primary sequence and secondary structure indicated that the addition of structural features to the sequence features can improve the accuracy and robustness of both models (**Fig S1B**). Therefore, in the final model of both human and mouse, we combined those features together to obtain a better performance. Next, to evaluate the performance of m6ASNP, 4-, 6-, 8- and 10-fold cross validations were performed on both the human and mouse models. In both species, the AUCs of all of the validations were close and larger than 0.84 (**Fig. 1B and Fig. 1D**), indicating that m6ASNP is an accurate and robust predictor. To further assess the prediction capability in unknown data, we then compared m6ASNP with the two other publicly available predictors, iRNA-Methyl and SRAMP, in the independent test set. As a result, the performance of m6ASNP was found to be superior to all other predictors in both the human and mouse models (**Fig. 1C and Fig. 1E**).

To balance the prediction accuracy, we selected three thresholds with high, medium and low stringencies for classification based on the evaluation result from 10-fold cross-validation. The high, medium and low thresholds were selected by controlling the false positive rate at 0.05, 0.1 and 0.15, respectively. **Table 1** presented the detail performance under these three selected thresholds. In general, the high threshold provides the most stringent criterion and is usually used in large-scale prediction. The medium threshold is a balanced criterion and may be appropriate for most cases. The low threshold is the loosest criterion. When users expect to retain as much potential sites as possible, this threshold would be the best option.

Usage of m6ASNP

In m6ASNP, a standard VCF format or a simplified tab delimitated file are supported as input data (**Fig. 2A**). As an example, we applied m6ASNP to the "common and clinical" variants VCF file obtained from ClinVar that contain 7,397 variants. The predicted m^6A-associated variants are presented in an interactive table (**Fig. 2B**). Out of 7397 variants, 206 are predicted to affect the m^6A modification, either functional gain or loss of modification. The web server will conduct a comprehensive annotation and statistical analysis for all the predicted m^6A-associated variants. The m^6A-associated variants from ClinVar are mainly enriched in enzyme binding and DNA binding GO molecular functions (**Fig. 2C**). The sequence logos are presented to show the changes of gained and lossed m^6A sites between the reference and mutant sequences (**Fig. 2D**). The "GGACU" motif is more obvious in mutant sequences compared to reference sequences for functional gain variants. While for functional loss variants, the "GGACU" motif is less noticeable in mutant sequences. A circos plot is presented to have an overview of all the m^6A-associated variants (**Fig. 2E**).

Characteristics of m^6A-associated variants predicted by m6ASNP

We further applied m6ASNP to all the variants in dbSNP. As a result, we obtained 133,394 functional gain and 214,884 functional loss m^6A-associated variants. Among these m^6A-associated variants, 6,235 located at/near the m^6A sites from miCLIP experiments and 55,381 located at/near the m^6A sites from MeRIP-Seq experiments. To characterize m^6A-associated variants predicted by m6ASNP, we performed a systematic comparison between m^6A-associated variants and non-m^6A-associated variants (non-m^6A variants). We found that m^6A-associated variants were enriched in protein-coding genes (dbSNP147, 95.77%; dbSNP146, 92.12%), and significantly concentrated in CDS and 3'UTR (**Fig. S2A, Table S1**). Interestingly, in both CDS and UTR region, m^6A-associated variants were more conserved than non-m^6A variants (**Fig. 3A**). For those conserved m^6A-associated variants, a significant portion was synonymous compared to all conserved variants (**Fig. 3B,** $p < 0.0001$, hypergeometric test). To further explain the functional role of m^6A-associated variants, we divided the predicted m^6A-associated variants into two groups: the functional gain and functional loss variants. The conservation analysis was performed on these two groups and the results were compared to non-m^6A variants in both CDS and UTR region (**Fig. S3**A). Strikingly, in most cases, the functional loss variants were found to be more conservative comparing to the gain variants, suggesting that the loss of existing

m^6A sites may undergo stronger selective pressure than the gain mutations on potential adenylate sites. Moreover, m^6A-associated variants were predicted to be more deleterious than non-m^6A variants in both the CDS and UTR region (**Fig. 3C**, two-tailed population test). Again, for the predicted data, the functional loss variants appeared to have a higher deleteriousness comparing to the functional gain variants and the non-m^6A variants (**Fig S3B**). Taken together, we conclude that m^6A-associated variants, especially the functional loss variants, may have important roles and could be driven by positive selection in mammalian genomes. Furthermore, there were more m^6A-associated variants located near the splice sites relative to the non-m^6A variants, mostly distributed in the 20-30bp flanking region of the splicing sites, implying that the variants were likely to affect RNA splicing as the means of changing the m^6A levels (**Fig. 3D**). Moreover, the m^6A-associated variants preferentially locate in genes with multiple transcripts (**Fig. S2B**). These results were in agreement with the findings reported by Xiao *et.al* [24].

m^6A-associated variants in disease

Genome wide association studies (GWAS) have revealed many disease-related variants. However, pathogenesis mechanism for most of these disease-related variants were still unknown. We found 1,919 m^6A-associated variants from human dbSNP were recorded either in GWAS studies or ClinVar database. These 1,919 m^6A-associated variants were related to various diseases, including cardiovascular phenotype, muscular dystrophy, Tuberous sclerosis syndrome and cancer. Among them, Hereditary cancer (436 variants, 22.74%, p=2.27e-30, Chi-squared test), Familial breast cancer (96 variants, 5.01%; p=8.33e-9, Chi-squared test) and Hereditary nonpolyposis colorectal cancer (73 variants, 3.81%; p=5.5e-5, Chi-squared test) were the top enriched disease types (**Table S2**). Our findings provided insights into the potential pathogenesis mechanism for many diseases related variants whose functions were not clear before.

Synonymous variants are neglected in most previous studies of disease. Since m6ASNP can be used to predict the effect of both non-synonymous and synonymous variant, this tool could significantly supplement the function of current annotating tools that mainly focus on non-synonymous variants. Indeed, among the m^6A-associated variants predicted by m6ASNP, 59.86% and 25.67% are synonymous variants in mouse dbSNP and human dbSNP, respectively. By using m6ASNP, we have identified many m^6A-associated synonymous variants that have been shown to be disease-related. For

instance, rs139362268, a synonymous variant of *PALB2*, is related to breast cancer and pancreatic cancer. Interestingly, we observed that rs139362268 was occurred in the m^{6}A site of *PALB2*, in which m^{6}A peaks were detected in six MeRIP-Seq experiments (**Fig. S4A**). We speculated that the cancer-related synonymous variant rs139362268 might be functional through dysregulation of m^{6}A modification.

m^{6}A-associated variants in post-transcriptional regulation

It has been reported that m^{6}A sites could recruit RBPs that play critical roles in post-transcriptional regulations [51]. We systematically examined the genomic position relationship between m^{6}A-associated variants and RBPs to determine whether m^{6}A-associated variants function through RBPs. We found the m^{6}A-associated variants were significantly enriched in RBP-binding regions compared to the non-m^{6}A variants (**Fig. S4B**). More than 50% of the human m^{6}A-associated variants located within RBP-binding regions. We found 19 RBPs were significantly overlapped with the regions having m^{6}A-associated variants (**Table S3**). As expected, the m^{6}A reader YTHDF2 and m^{6}A eraser ALKBH5 were significantly overlapped with the regions having m^{6}A-associated variants compared to the randomly selected regions. Moreover, GO annotations demonstrated that these RBPs are enriched in RNA splicing, RNA translation and miRNA regulation (**Table S3**). Among them, SFRS1, a known splicing factor, is reportedly involved in alternative splicing and colocalized with ALKBH5 in a demethylation-dependent manner, suggesting it might be participated in the regulation of RNA methylation [52].

It has been reported that m^{6}A sites are enriched in miRNA target sites and regulated by miRNAs [53]. Consistent with this, we found m^{6}A-associated variants predicted by m6ASNP occurred significantly more frequently in miRNA target sites than the non-m^{6}A variants (**Fig. S4C**). The miRNAs with a significant number of m^{6}A-associated variants were listed in **Table S4.** Among them, *miR-132-3p* and *miR-212-3p* were mainly expressed in the brain and played critical roles in neuronal functions as well as circadian clock entrainment [54], which is consistent with m^{6}A function [55]. Interestingly, m^{6}A-associated variants related to *miR-132-3p* and *miR-212-3p* were identified in both human and mouse, suggesting a conservation of function in these variants.

Discussion

There is growing evidence showing that aberrant m^6A modification is a potential pathogenesis mechanism in many diseases including cancer, which suggests the variants disrupting m^6A modification might cause diseases. However, currently there is still lack of methodology for annotating variants from high-throughput sequencing studies by m^6A function. To address this, we have developed a novel computation model named m6ASNP that is dedicated to predict variants disrupting m^6A modification. Using m6ASNP, we performed further functional analysis on m^6A-associated variants. By integrating data set regarding RBP-binding regions, miRNA-targets and splicing sites, m6ASNP can help to reveal the potential relationship among variants, m^6A modification and other post-transcriptional regulation. Also, the disease-association analysis had identified more than 2,000 disease-related variants that may be linked with alterations of m^6A modification. This finding further proved that m6ASNP is a promising tool for studying the potential role of m^6A variants in clinical investigation.

In conclusion, m6ASNP is a useful computational webserver for annotating variants by m^6A function. m6ASNP will serve as a supplemental method to run in parallel with other annotating tools to comprehensively predicting the function of the variants, for both synonymous and non-synonymous, in the high-throughput sequencing studies of diseases.

Methods

Construction of m^6A site prediction model

The sequences of the flanking regions 30 nucleotides upstream and downstream of a given m^6A residue were extracted. To transform the primary sequences to numeric vectors, each nucleotide was encoded by four distinct variables. In total, 60 numeric variables were generated for a single m^6A residue. As reported in recent studies [56, 57], specific RNA secondary structures around the potential adenosines can affect the enzymatic process of RNA methylation. We therefore added secondary structure features to our prediction model. Using the Nussinov algorithm [58], we first predicted the secondary structure for each m^6A residue and marked the structure state (paired or not paired) with a bracket or dot. For example, a given m^6A nucleotide with the sequence TTCCGGGACTGGCAGG could be represented as ((((())))((.())).. Next, we extracted the secondary structure triplet, formed by the structure state of the three adjacent nucleotides obtained from the predicted RNA structure. The

number of occurrences of each triplet in the sequence was counted and normalized to produce a 27-dimension feature vector. Combining all the primary sequences and secondary structure features, we constructed an 87-dimension vector for each m^6A residue. These vectors were subsequently used as the input for a random forest classifier for training and prediction.

The random forest classifier for human and mouse were train separately on the above collected training set. The tree number was optimized as 500 and the features used for each splitting were set to 9. To assess the performance, we employed 4, 6, 8, 10-fold cross-validation on the training set. The additional test set was also applied in our study to evaluate the robustness. The *sensitivity*, *specificity* and *Matthew's correlation coefficient* were used to measure the predictor's performance.

Construction of m6ASNP

Based on the m^6A site prediction model, we then developed a computational pipeline to predict the effect of variants on m^6A modification. Firstly, variants were mapped to known transcripts. The wild-type and mutant form of the transcript sequences were then generated for m^6A site prediction. For an m^6A site that occurred in the wild-type transcript and disrupted in the mutant transcript, we defined it as an m^6A-associated loss variant. The m^6A-associated gain variant is conversely formed. To measure the altered degree of m^6A modifications, equation 1 was defined as shown below.

$$S=ln\left(\frac{RF\_Score_{wild\text{-}type}}{RF\_Score_{mutant}}\right) \qquad \text{Equation 1}$$

In the above equation, S denoted as the alteration score which quantitatively represented the degree of m^6A alterations between reference and mutant samples. *RF_Score* is the predicted score of a given m^6A site from the random forest model. Obviously, the alteration scores larger than 0 represented m^6A-gain alterations, while score lower than 0 represented m^6A-loss alterations. In some m^6A-associated loss variants, alteration scores were assigned to MAX, which mean that the core AC motif is destroyed by genetic variants and leading to complete losses of m^6A at those sites.

To provide convenience to the research community we developed a web server called "m6ASNP" to specifically predict the effect of variants on m^6A modification. m6ASNP was implemented using JAVA

and PHP, and is freely accessible at http://m6asnp.renlab.org.

Derivation of the m6A-associated variants

Based on miCLIP-seq, PA-m^{6}A-seq and MeRIP-seq data, we then combined them with the SNV data from dbSNP and performed m^{6}A-association prediction using m6ASNP. Following the same procedure proposed in our previously published work [59], we constructed three confidence levels of annotations of m^{6}A-associated variants for subsequent analysis.

The first annotation was the high confidence level data that contained the m^{6}A-associated variants derived from miCLIP-seq and PA-m^{6}A-seq experiments. Notably, the PA-m^{6}A-seq can only detect m^{6}A signal in a resolution of ~23nt, therefore, to obtain precise modification sites, we scanned through all the peak regions and extracted adenosine sites that conformed to DRACH motif as final m^{6}A sites. On this basis, we retained the variants that located nearby the m^{6}A sites as the m^{6}A-associated variants.

The second annotation was the medium confidence level data. We first downloaded all the published MeRIP-seq data from the GEO database. According to the standard analysis pipeline for MeRIP-seq data, we applied MACS2 [60], MeTPeak [61] and Meyer's method [62] to identify the m^{6}A peaks in each study separately. Generally speaking, in MeRIP-seq experiments, if a given region is identified as enriched in most of the adopted methods, it is more likely to be a true modification signal. Therefore, to obtain reliable m^{6}A peaks, a tool called MSPC [63] was then applied to construct consensus peaks from the above three methods. In those consensus peaks, we then applied m6ASNP to predict m^{6}A-associated variants that significantly change the DRACH motif.

The third annotation was the low confidence level data, where we used the whole transcriptome sequences for prediction. With a high threshold, m6ASNP will predict the potential m^{6}A-associated variants from all collected genetic variants.

In summary, we had constructed 13,703 high confidence level, 54,222 medium confidence level and 243,880 low confidence level of m^{6}A-associated variants for human. Another 935 high confidence level, 9,404 medium confidence level and 17,739 low confidence level data were also constructed for mouse.

Annotation of m^6A-associated variants

All the identified m^6A-associated variants were annotated by the transcript structure, including the CDS, 3’ UTR, 5’ UTR, start codon and stop codon etc. For the annotation of non-coding RNA DASHR [64], miRBase(version 21)(miRBase, RRID:SCR_003152) [65], GtRNAdb [66] and piRNABank [67] were used. To test whether the m^6A-associated variants were more preferentially distributed in specific transcript structures, we calculated the proportion of variants that located in a given transcript structure. In order to avoid bias, only the variants that were annotated in mRNA were used, and the proportion in 5’-UTR, CDS and 3’-UTR were calculated. A two-tailed proportion test was then adopted to compare the proportion difference between m^6A-associated variants and non-m^6A variants. Besides, to evaluate their conservation scores and deleteriousness, we further annotated the m^6A-associated variants by ANNOVAR (updated to 1 February 2016) (ANNOVAR, RRID:SCR_012821) [68]. To avoid any bias, we only preserved those variants located in mRNA for analysis, and compared the conservative and deleterious differences between m^6A-associated variants and non-m^6A variants in the same exon. As the selective pressures were quite different in protein-coding sequences and untranslated regions, the above comparison was carried out separately for the CDS and UTR regions. Specifically, the conservation scores were calculated by phastCons with 100-way and 60-way gene conservation profiles for the human and mouse respectively [69]. The deleteriousness of each variants was measured by integrating the prediction results from five pieces of software (SIFT [70], PolyPhen2 HVAR [10], PolyPhen2 HDIV [10], LRT [71] and FATHMM [72]). We defined an aggregate score by counting the number of the above methods that consider an SNV to be deleterious. A deleterious score of 0 means that the variant is predicted to be tolerated in all methods, while for a deleterious score of 5 means that the corresponding variant is predicted to be deleterious in all five predictors. As a result, the aggregate score may range from 0 to 5, and a higher score indicate a higher probability of deleterious.

Disease association analysis

An LD analysis was performed for each GWAS disease-associated SNP. We used Haploview (Haploview, RRID:SCR_003076) to obtain the LD mutations using a parameter $r^2 > 0.8$ in at least one of the four populations from CHB, CEU, JPT and TSI. Then, we selected all m^6A-associated variants

by mapping the variants to GWAS disease-associated SNPs and their LD mutations. Moreover, we collected ClinVar data to annotate the m^{6}A-associated variants with specific functions.

Post-transcriptional regulation association analysis

First, the m^{6}A-associated variants were intersected with the collected RNA-binding protein (RBP) regions for the same sample. We matched all m^{6}A-associated variants with miRNA targets to obtain the m^{6}A-associated variants that potentially impacted the miRNA-target interactions. Additionally, we extracted 100 base pairs (bp) upstream of the 5' splicing sites and 100 bp downstream of the 3' splicing sites. Subsequently, we matched the m^{6}A-associated variants to these regions to obtain the splicing sites affected by the m^{6}A-associated variants.

Identification of significant RBPs and miRNAs

To evaluate whether the m^{6}A-associated variants were significantly enriched in RBP regions, an empirical evaluation was performed for each RBP. Using YTHDF2 as an example, the process may be described as follows.

First, we calculated the number of m^{6}A-associated variants within the YTHDF2 binding regions (defined as N_{RBP}). Second, because certain m^{6}A-associated variants randomly occur within the YTHDF2 binding regions, we estimated the background count of m^{6}A-associated variants for YTHDF2 (defined as N_B). Thus, we extracted the longest transcript for each gene from the gene annotation files. The weight of the *i*th gene was defined as below:

$$w(i)=\frac{L(i)}{\sum_{i=0}^{n} L(i)} \qquad \text{Equation 2}$$

$$\sum_{i=0}^{n} w(i)=1 \qquad \text{Equation 3}$$

where n was the total number of genes annotated, and $L(i)$ was the length (bp) of the *i*th gene. Then, we extracted the same-length reads of all YTHDF2-binding regions, which was defined as N_B, using weighted random sampling of all transcripts collected above. We repeated this procedure 50,000 times and then obtained the frequency F_{RBP} when N_B was greater than N_{RBP} in the cycle. This frequency may be regarded as an estimation of the probability that observing N_B greater then N_{RBP} in random condition. Next, the Benjamini-Hochberg method was applied to control the false positives. An adjusted F_{RBP} less

than 0.05 was considered a small probability event, suggesting that the m^{6}A-associated variants were more likely to occur in the RBP-binding regions of YTHDF2. All significant RBPs are listed in **Table S2**. Certain significant miRNAs, which are listed in **Table S3**, were obtained by performing a similar analysis of miRNA targets.

Availability of supporting source code and requirements

Project name: m6ASNP

Project home page: https://m6asnp.renlab.org

https://github.com/RenLabBioinformatics/m6ASNP

RRID: SCR_016048

Operating system(s): platform independent

Programing language: PHP, java, javascript

License: GPLv3

Availability of supporting data

The training data and test data collected from Linder *et al.* and Ke *et al.* are available in the supplementary data. These and snapshots of the code are also available from the *GigaScience* GigaDB repository[73].

Declarations

Abbreviations:

m^{6}A: N6-methyladenosine

SNP: single nucleotide polymorphism

SVM: support vector machine

AUC: area under curve

VCF: Variant call format

GO: Gene ontology

LD: Linkage disequilibrium

GWAS: Genome-wide association study

RBP: RNA binding protein

Ethics approval and consent to participate

Not applicable

Disclosure statement

The author(s) declare that they have no competing interests

Funding

This work was supported by grants from the National Key Research and Development Program [2017YFA0106700]; National Natural Science Foundation of China [31771462, 81772614, 31471252, 31500813 and U1611261]; Guangdong Natural Science Foundation [2014TQ01R387, 2014A030313181 and 2017A030313134]; China Postdoctoral Science Foundation [2017M622864]; Fundamental Research Funds for the Central Universities [No. 17lgpy106].

Authors' contributions

ZZ and JR conceived, designed, and supervised all phases of the project. YX and SJ developed the prediction model. YX and ZH designed and implemented the Web server. YZ, ML and DP performed data analysis. ZZ, YX, SJ and JR wrote the manuscript. All authors read and approved the final manuscript.

References

1. Carvalho S, Catarino TA, Dias AM, Kato M, Almeida A, Hessling B, et al. Preventing E-cadherin aberrant N-glycosylation at Asn-554 improves its critical function in gastric cancer. Oncogene. 2016;35 13:1619-31. doi:10.1038/onc.2015.225.

2. Gonfloni S, Williams JC, Hattula K, Weijland A, Wierenga RK and Superti-Furga G. The role of the linker between the SH2 domain and catalytic domain in the regulation and function of Src. The EMBO journal. 1997;16 24:7261-71. doi:10.1093/emboj/16.24.7261.

3. Selezneva AI, Walden WE and Volz KW. Nucleotide-specific recognition of iron-responsive elements by iron regulatory protein 1. Journal of molecular biology. 2013;425 18:3301-10. doi:10.1016/j.jmb.2013.06.023.

4. Zhang B, Deng L, Qian Q, Xiong G, Zeng D, Li R, et al. A missense mutation in the

transmembrane domain of CESA4 affects protein abundance in the plasma membrane and results in abnormal cell wall biosynthesis in rice. Plant molecular biology. 2009;71 4-5:509-24. doi:10.1007/s11103-009-9536-4.

5. Heald R and McKeon F. Mutations of phosphorylation sites in lamin A that prevent nuclear lamina disassembly in mitosis. Cell. 1990;61 4:579-89.

6. Xu Y, Gray A, Hardie DG, Uzun A, Shaw S, Padbury J, et al. A novel, de novo mutation in the PRKAG2 gene: infantile-onset phenotype and the signaling pathway involved. American journal of physiology Heart and circulatory physiology. 2017;313 2:H283-H92. doi:10.1152/ajpheart.00813.2016.

7. McCabe MT, Graves AP, Ganji G, Diaz E, Halsey WS, Jiang Y, et al. Mutation of A677 in histone methyltransferase EZH2 in human B-cell lymphoma promotes hypertrimethylation of histone H3 on lysine 27 (H3K27). Proceedings of the National Academy of Sciences of the United States of America. 2012;109 8:2989-94. doi:10.1073/pnas.1116418109.

8. Liu X, Gao J, Sun Y, Zhang D, Liu T, Yan Q, et al. Mutation of N-linked glycosylation in EpCAM affected cell adhesion in breast cancer cells. Biological Chemistry. 2017;398 10:1119-26. doi:10.1515/hsz-2016-0232.

9. Sim NL, Kumar P, Hu J, Henikoff S, Schneider G and Ng PC. SIFT web server: predicting effects of amino acid substitutions on proteins. Nucleic acids research. 2012;40 Web Server issue:W452-7. doi:10.1093/nar/gks539.

10. Adzhubei IA, Schmidt S, Peshkin L, Ramensky VE, Gerasimova A, Bork P, et al. A method and server for predicting damaging missense mutations. Nature Methods. 2010;7 4:248-9. doi:10.1038/nmeth0410-248.

11. Ren J, Jiang C, Gao X, Liu Z, Yuan Z, Jin C, et al. PhosSNP for systematic analysis of genetic polymorphisms that influence protein phosphorylation. Molecular & cellular proteomics : MCP. 2010;9 4:623-34. doi:10.1074/mcp.M900273-MCP200.

12. Wagih O, Reimand J and Bader GD. MIMP: predicting the impact of mutations on kinase-substrate phosphorylation. Nature Methods. 2015;12 6:531-3. doi:10.1038/nmeth.3396.

13. Supek F, Minana B, Valcarcel J, Gabaldon T and Lehner B. Synonymous mutations frequently act as driver mutations in human cancers. Cell. 2014;156 6:1324-35.

doi:10.1016/j.cell.2014.01.051.

14. Sauna ZE and Kimchi-Sarfaty C. Understanding the contribution of synonymous mutations to human disease. Nature Reviews Genetics. 2011;12 10:683-91. doi:10.1038/nrg3051.

15. Parmley JL, Chamary JV and Hurst LD. Evidence for purifying selection against synonymous mutations in mammalian exonic splicing enhancers. Molecular biology and evolution. 2006;23 2:301-9. doi:10.1093/molbev/msj035.

16. Chamary JV and Hurst LD. Evidence for selection on synonymous mutations affecting stability of mRNA secondary structure in mammals. Genome Biology. 2005;6 9:R75. doi:10.1186/gb-2005-6-9-r75.

17. Drummond DA and Wilke CO. Mistranslation-induced protein misfolding as a dominant constraint on coding-sequence evolution. Cell. 2008;134 2:341-52. doi:10.1016/j.cell.2008.05.042.

18. Roundtree IA, Evans ME, Pan T and He C. Dynamic RNA Modifications in Gene Expression Regulation. Cell. 2017;169 7:1187-200. doi:10.1016/j.cell.2017.05.045.

19. Feigerlova E and Battaglia-Hsu SF. Role of post-transcriptional regulation of mRNA stability in renal pathophysiology: focus on chronic kidney disease. FASEB journal : official publication of the Federation of American Societies for Experimental Biology. 2017;31 2:457-68. doi:10.1096/fj.201601087RR.

20. Kiebler MA, Scheiffele P and Ule J. What, where, and when: the importance of post-transcriptional regulation in the brain. Frontiers in neuroscience. 2013;7:192. doi:10.3389/fnins.2013.00192.

21. Mort M, Sterne-Weiler T, Li B, Ball EV, Cooper DN, Radivojac P, et al. MutPred Splice: machine learning-based prediction of exonic variants that disrupt splicing. Genome Biology. 2014;15 1:R19. doi:10.1186/gb-2014-15-1-r19.

22. Pruesse E, Quast C, Knittel K, Fuchs BM, Ludwig W, Peplies J, et al. SILVA: a comprehensive online resource for quality checked and aligned ribosomal RNA sequence data compatible with ARB. Nucleic acids research. 2007;35 21:7188-96. doi:10.1093/nar/gkm864.

23. Fu Y, Dominissini D, Rechavi G and He C. Gene expression regulation mediated through reversible m(6)A RNA methylation. Nature Reviews Genetics. 2014;15 5:293-306. doi:10.1038/nrg3724.

24. Xiao W, Adhikari S, Dahal U, Chen YS, Hao YJ, Sun BF, et al. Nuclear m(6)A Reader YTHDC1 Regulates mRNA Splicing. Molecular cell. 2016;61 4:507-19. doi:10.1016/j.molcel.2016.01.012.

25. Wang Y, Li Y, Toth JI, Petroski MD, Zhang Z and Zhao JC. N6-methyladenosine modification destabilizes developmental regulators in embryonic stem cells. Nature Cell Biology. 2014;16 2:191-8. doi:10.1038/ncb2902.

26. Meyer KD, Patil DP, Zhou J, Zinoviev A, Skabkin MA, Elemento O, et al. 5' UTR m(6)A Promotes Cap-Independent Translation. Cell. 2015;163 4:999-1010. doi:10.1016/j.cell.2015.10.012.

27. Boissel S, Reish O, Proulx K, Kawagoe-Takaki H, Sedgwick B, Yeo GS, et al. Loss-of-function mutation in the dioxygenase-encoding FTO gene causes severe growth retardation and multiple malformations. American journal of human genetics. 2009;85 1:106-11. doi:10.1016/j.ajhg.2009.06.002.

28. Daoud H, Zhang D, McMurray F, Yu A, Luco SM, Vanstone J, et al. Identification of a pathogenic FTO mutation by next-generation sequencing in a newborn with growth retardation and developmental delay. Journal of medical genetics. 2016;53 3:200-7. doi:10.1136/jmedgenet-2015-103399.

29. Jonkhout N, Tran J, Smith MA, Schonrock N, Mattick JS and Novoa EM. The RNA modification landscape in human disease. RNA (New York, NY). 2017; doi:10.1261/rna.063503.117.

30. McGuinness DH and McGuinness D. m6a RNA methylation: the implications for health and disease. Journal of Cancer Science and Clinical Oncology. 2014;1 1 doi:10.15744/2394-6520.1.105.

31. Fawcett KA and Barroso I. The genetics of obesity: FTO leads the way. Trends in genetics : TIG. 2010;26 6:266-74. doi:10.1016/j.tig.2010.02.006.

32. Li Z, Weng H, Su R, Weng X, Zuo Z, Li C, et al. FTO Plays an Oncogenic Role in Acute Myeloid Leukemia as a N6-Methyladenosine RNA Demethylase. Cancer cell. 2017;31 1:127-41. doi:10.1016/j.ccell.2016.11.017.

33. Lewis SJ, Murad A, Chen L, Davey Smith G, Donovan J, Palmer T, et al. Associations between an obesity related genetic variant (FTO rs9939609) and prostate cancer risk. PloS

one. 2010;5 10:e13485. doi:10.1371/journal.pone.0013485.

34. Zhang C, Samanta D, Lu H, Bullen JW, Zhang H, Chen I, et al. Hypoxia induces the breast cancer stem cell phenotype by HIF-dependent and ALKBH5-mediated m(6)A-demethylation of NANOG mRNA. Proceedings of the National Academy of Sciences of the United States of America. 2016;113 14:E2047-56. doi:10.1073/pnas.1602883113.

35. Zhang C, Zhi WI, Lu H, Samanta D, Chen I, Gabrielson E, et al. Hypoxia-inducible factors regulate pluripotency factor expression by ZNF217- and ALKBH5-mediated modulation of RNA methylation in breast cancer cells. Oncotarget. 2016;7 40:64527-42. doi:10.18632/oncotarget.11743.

36. Zhang Z, Zhang G, Kong C, Zhan B, Dong X and Man X. METTL13 is downregulated in bladder carcinoma and suppresses cell proliferation, migration and invasion. Scientific reports. 2016;6:19261. doi:10.1038/srep19261.

37. Ma JZ, Yang F, Zhou CC, Liu F, Yuan JH, Wang F, et al. METTL14 suppresses the metastatic potential of hepatocellular carcinoma by modulating N6 -methyladenosine-dependent primary MicroRNA processing. Hepatology (Baltimore, Md). 2017;65 2:529-43. doi:10.1002/hep.28885.

38. Chen W, Feng P, Ding H, Lin H and Chou KC. iRNA-Methyl: Identifying N(6)-methyladenosine sites using pseudo nucleotide composition. Analytical biochemistry. 2015;490:26-33. doi:10.1016/j.ab.2015.08.021.

39. Liu Z, Xiao X, Yu DJ, Jia J, Qiu WR and Chou KC. pRNAm-PC: Predicting N(6)-methyladenosine sites in RNA sequences via physical-chemical properties. Analytical biochemistry. 2016;497:60-7. doi:10.1016/j.ab.2015.12.017.

40. Zhou Y, Zeng P, Li YH, Zhang Z and Cui Q. SRAMP: prediction of mammalian N6-methyladenosine (m6A) sites based on sequence-derived features. Nucleic acids research. 2016;44 10:e91. doi:10.1093/nar/gkw104.

41. Linder B, Grozhik AV, Olarerin-George AO, Meydan C, Mason CE and Jaffrey SR. Single-nucleotide-resolution mapping of m6A and m6Am throughout the transcriptome. Nature methods. 2015;12 8:767-72. doi:10.1038/nmeth.3453.

42. Ke S, Alemu EA, Mertens C, Gantman EC, Fak JJ, Mele A, et al. A majority of m6A residues are in the last exons, allowing the potential for 3' UTR regulation. Genes & development.

2015;29 19:2037-53. doi:10.1101/gad.269415.115.

43. Chen K, Lu Z, Wang X, Fu Y, Luo GZ, Liu N, et al. High-resolution N(6) -methyladenosine (m(6) A) map using photo-crosslinking-assisted m(6) A sequencing. Angewandte Chemie (International ed in English). 2015;54 5:1587-90. doi:10.1002/anie.201410647.

44. Li JH, Liu S, Zhou H, Qu LH and Yang JH. starBase v2.0: decoding miRNA-ceRNA, miRNA-ncRNA and protein-RNA interaction networks from large-scale CLIP-Seq data. Nucleic acids research. 2014;42 Database issue:D92-7. doi:10.1093/nar/gkt1248.

45. Yang YC, Di C, Hu B, Zhou M, Liu Y, Song N, et al. CLIPdb: a CLIP-seq database for protein-RNA interactions. BMC genomics. 2015;16:51. doi:10.1186/s12864-015-1273-2.

46. Welter D, MacArthur J, Morales J, Burdett T, Hall P, Junkins H, et al. The NHGRI GWAS Catalog, a curated resource of SNP-trait associations. Nucleic acids research. 2014;42 Database issue:D1001-6. doi:10.1093/nar/gkt1229.

47. Johnson AD and O'Donnell CJ. An open access database of genome-wide association results. BMC medical genetics. 2009;10:6. doi:10.1186/1471-2350-10-6.

48. Mailman MD, Feolo M, Jin Y, Kimura M, Tryka K, Bagoutdinov R, et al. The NCBI dbGaP database of genotypes and phenotypes. Nature genetics. 2007;39 10:1181-6. doi:10.1038/ng1007-1181.

49. Becker KG, Barnes KC, Bright TJ and Wang SA. The genetic association database. Nature genetics. 2004;36 5:431-2. doi:10.1038/ng0504-431.

50. Landrum MJ, Lee JM, Benson M, Brown G, Chao C, Chitipiralla S, et al. ClinVar: public archive of interpretations of clinically relevant variants. Nucleic acids research. 2016;44 D1:D862-8. doi:10.1093/nar/gkv1222.

51. Liu J, Yue Y, Han D, Wang X, Fu Y, Zhang L, et al. A METTL3-METTL14 complex mediates mammalian nuclear RNA N6-adenosine methylation. Nature chemical biology. 2014;10 2:93-5. doi:10.1038/nchembio.1432.

52. Zheng G, Dahl JA, Niu Y, Fedorcsak P, Huang CM, Li CJ, et al. ALKBH5 is a mammalian RNA demethylase that impacts RNA metabolism and mouse fertility. Molecular cell. 2013;49 1:18-29. doi:10.1016/j.molcel.2012.10.015.

53. Chen T, Hao YJ, Zhang Y, Li MM, Wang M, Han W, et al. m(6)A RNA methylation is regulated by microRNAs and promotes reprogramming to pluripotency. Cell stem cell.

2015;16 3:289-301. doi:10.1016/j.stem.2015.01.016.

54. Wanet A, Tacheny A, Arnould T and Renard P. miR-212/132 expression and functions: within and beyond the neuronal compartment. Nucleic acids research. 2012;40 11:4742-53. doi:10.1093/nar/gks151.

55. Fustin JM, Doi M, Yamaguchi Y, Hida H, Nishimura S, Yoshida M, et al. RNA-methylation-dependent RNA processing controls the speed of the circadian clock. Cell. 2013;155 4:793-806. doi:10.1016/j.cell.2013.10.026.

56. Roost C, Lynch SR, Batista PJ, Qu K, Chang HY and Kool ET. Structure and thermodynamics of N6-methyladenosine in RNA: a spring-loaded base modification. Journal of the American Chemical Society. 2015;137 5:2107-15. doi:10.1021/ja513080v.

57. Cao G, Li HB, Yin Z and Flavell RA. Recent advances in dynamic m6A RNA modification. Open biology. 2016;6 4:160003. doi:10.1098/rsob.160003.

58. Eddy SR. How do RNA folding algorithms work? Nature biotechnology. 2004;22 11:1457-8. doi:10.1038/nbt1104-1457.

59. Zheng Y, Nie P, Peng D, He Z, Liu M, Xie Y, et al. m6AVar: a database of functional variants involved in m6A modification. Nucleic acids research. 2018;46 D1:D139-D45. doi:10.1093/nar/gkx895.

60. Zhang Y, Liu T, Meyer CA, Eeckhoute J, Johnson DS, Bernstein BE, et al. Model-based analysis of ChIP-Seq (MACS). Genome Biology. 2008;9 9:R137. doi:10.1186/gb-2008-9-9-r137.

61. Cui X, Meng J, Zhang S, Chen Y and Huang Y. A novel algorithm for calling mRNA m6A peaks by modeling biological variances in MeRIP-seq data. Bioinformatics (Oxford, England). 2016;32 12:i378-i85. doi:10.1093/bioinformatics/btw281.

62. Meyer KD, Saletore Y, Zumbo P, Elemento O, Mason CE and Jaffrey SR. Comprehensive analysis of mRNA methylation reveals enrichment in 3' UTRs and near stop codons. Cell. 2012;149 7:1635-46. doi:10.1016/j.cell.2012.05.003.

63. Jalili V, Matteucci M, Masseroli M and Morelli MJ. Using combined evidence from replicates to evaluate ChIP-seq peaks. Bioinformatics (Oxford, England). 2015;31 17:2761-9. doi:10.1093/bioinformatics/btv293.

64. Leung YY, Kuksa PP, Amlie-Wolf A, Valladares O, Ungar LH, Kannan S, et al. DASHR:

database of small human noncoding RNAs. Nucleic acids research. 2016;44 D1:D216-22. doi:10.1093/nar/gkv1188.

65. Kozomara A and Griffiths-Jones S. miRBase: annotating high confidence microRNAs using deep sequencing data. Nucleic acids research. 2014;42 Database issue:D68-73. doi:10.1093/nar/gkt1181.

66. Chan PP and Lowe TM. GtRNAdb 2.0: an expanded database of transfer RNA genes identified in complete and draft genomes. Nucleic acids research. 2016;44 D1:D184-9. doi:10.1093/nar/gkv1309.

67. Sai Lakshmi S and Agrawal S. piRNABank: a web resource on classified and clustered Piwi-interacting RNAs. Nucleic acids research. 2008;36 Database issue:D173-7. doi:10.1093/nar/gkm696.

68. Wang K, Li M and Hakonarson H. ANNOVAR: functional annotation of genetic variants from high-throughput sequencing data. Nucleic acids research. 2010;38 16:e164. doi:10.1093/nar/gkq603.

69. Siepel A, Bejerano G, Pedersen JS, Hinrichs AS, Hou M, Rosenbloom K, et al. Evolutionarily conserved elements in vertebrate, insect, worm, and yeast genomes. Genome research. 2005;15 8:1034-50. doi:10.1101/gr.3715005.

70. Kumar P, Henikoff S and Ng PC. Predicting the effects of coding non-synonymous variants on protein function using the SIFT algorithm. Nature Protocols. 2009;4 7:1073-81. doi:10.1038/nprot.2009.86.

71. Chun S and Fay JC. Identification of deleterious mutations within three human genomes. Genome research. 2009;19 9:1553-61. doi:10.1101/gr.092619.109.

72. Shihab HA, Gough J, Cooper DN, Stenson PD, Barker GL, Edwards KJ, et al. Predicting the functional, molecular, and phenotypic consequences of amino acid substitutions using hidden Markov models. Human mutation. 2013;34 1:57-65. doi:10.1002/humu.22225.

73. Jiang S; Xie Y; He Z; Zhang Y; Zhao Y; Chen L; Zheng Y; Miao Y; Zuo Z; Ren J (2018): Supporting data for "m6ASNP: a tool for annotating genetic variants by m6A function" GigaScience Database. http://dx.doi.org/10.5524/100428

74. Cui Y, Chen X, Luo H, Fan Z, Luo J, He S, et al. BioCircos.js: an interactive Circos JavaScript library for biological data visualization on web applications. Bioinformatics

(Oxford, England). 2016;32 11:1740-2. doi:10.1093/bioinformatics/btw041.

Figure legends

Fig. 1 - The construction of m6ASNP. (A) The computational pipeline for identifying m^{6}A-associatied variants. (1) The single-nucleotide-resolution data were collected from recently published miCLIP-seq experiments. (2) The primary sequence and secondary structure features were extracted for subsequent model training process. (3) Genetic variants, such as somatic variants or germline SNPs, were inputted into the computation pipeline. (4) The flanking sequence around the potential m^{6}A residue were constructed for both wild-type and mutant samples based on the inputted variants. (5) The loss and gain variants were predicted according to the above data. (B) 4, 6, 8, 10-fold cross-validation were performed on the human model. (C) The performance comparison between m6ASNP and other state-of-art tools on the human test set. (D) The evaluation results of 4, 6, 8, 10-fold cross-validation in mouse model. (E) The performance comparison between m6ASNP and other state-of-art tools on the mouse test set.

Fig. 2 - A snapshot of m6ASNP webserver. (A) The main interface. Variants can be inputted as standard VCF format or tab-delimited flat format. A file uploading module was implemented to support large-scale prediction of m^{6}A-associated variants. (B) The prediction results were listed in the interactive table which allowing fast retrieval of the result data. (C) The Gene Ontology annotation were performed on the predicted m^{6}A-associated variants. (D) To present the alterations of m^{6}A motif, the sequence logos were generated automatically for both functional gain and loss variants. (E) The gain and loss m^{6}A-associated variants, as well as the original SNPs, were illustrated in the circos plot at a genomic level by the BioCircos [74] library.

Fig. 3 - Characteristics of m^{6}A-associated variants predicted by m6ASNP. (A) The cumulative distribution function (CDF) of phastCons score for different levels of m^{6}A-associated variants and non-m^{6}A variants in mouse dbSNP and human dbSNP. (B) Proportional distribution of different variant types for the conserved m^{6}A-associated variants. (C) Proportional distribution of the m^{6}A-associated variants and non-m^{6}A variants at three deleterious levels predicted by a combination

of five variant function predictors. A two-tailed test of the population proportion was used to assess significance. (D) Proportional distribution of m^6A-associated variants and non-m^6A variants at different distances from the splicing sites.

Tables

Table 1 – The prediction performance from 10-fold cross-validation under the high, medium and low threshold.

**Threshold**	**Human**					**Mouse**				
	Ac	***Sn***	***Sp***	***MCC***	***Pr***	***Ac***	***Sn***	***Sp***	***MCC***	***Pr***
**High**	0.7235	0.2781	0.9461	0.3158	0.7208	0.7154	0.2477	0.9492	0.2894	0.7092
**Medium**	0.7487	0.4497	0.8981	0.3973	0.6882	0.7465	0.4467	0.8964	0.3918	0.6832
**Low**	0.7589	0.5837	0.8465	0.4439	0.6554	0.7591	0.5956	0.8409	0.4471	0.6518

Figure 1

Click here to download Figure Figure 1.tif

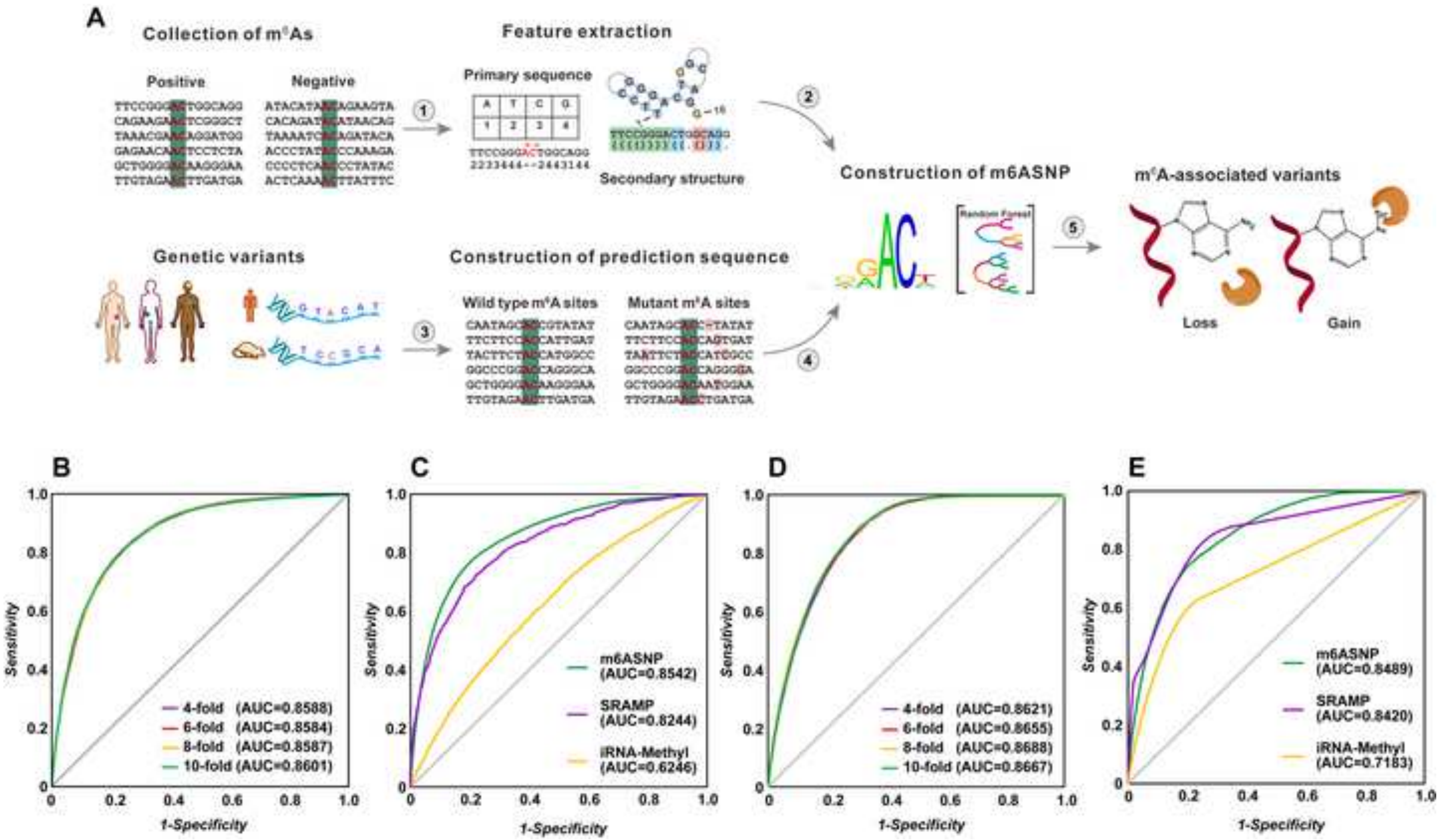

Figure 2 Click here to download Figure Figure 2.tif

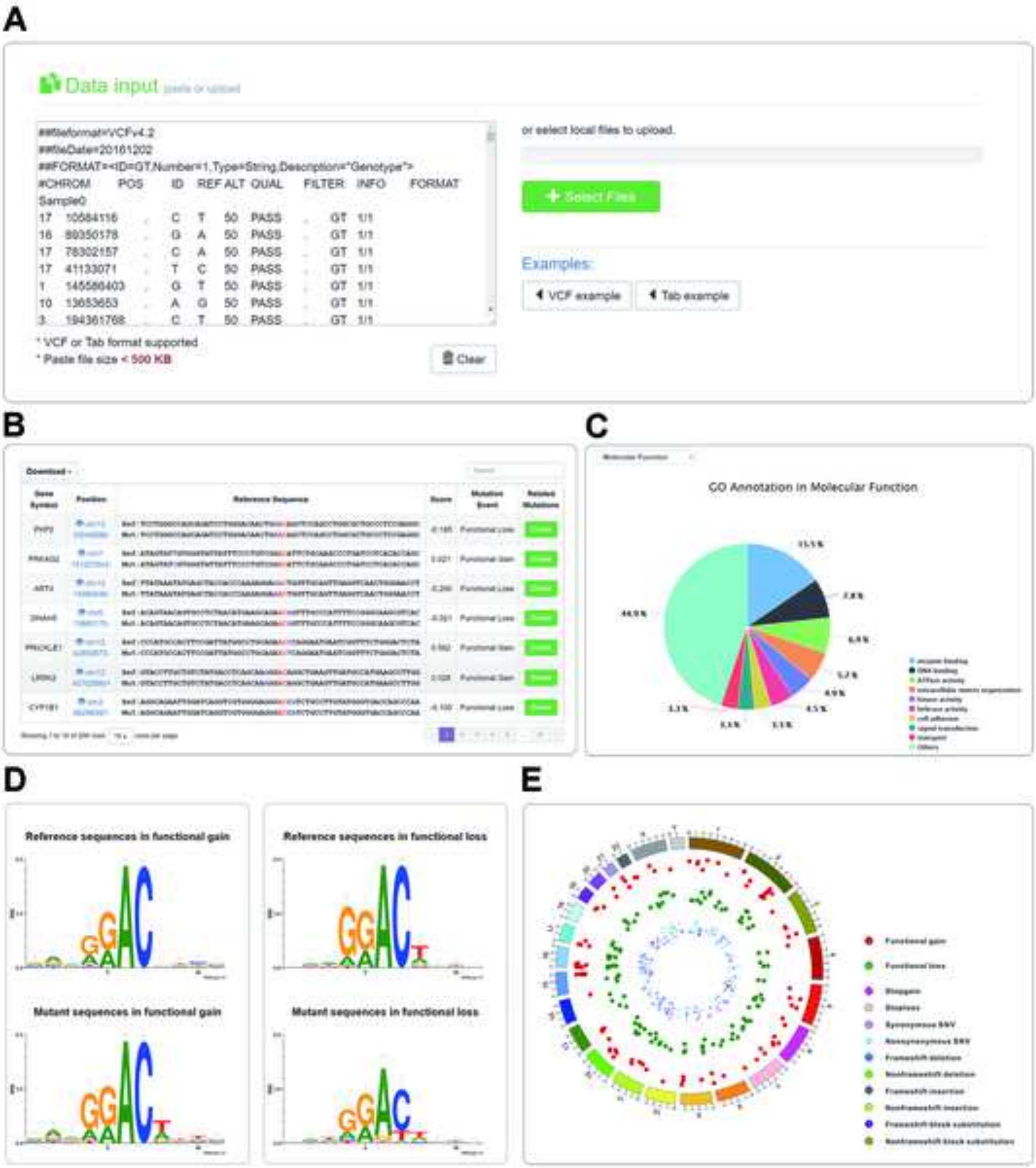

Figure 3

Click here to download Figure Figure 3.tif

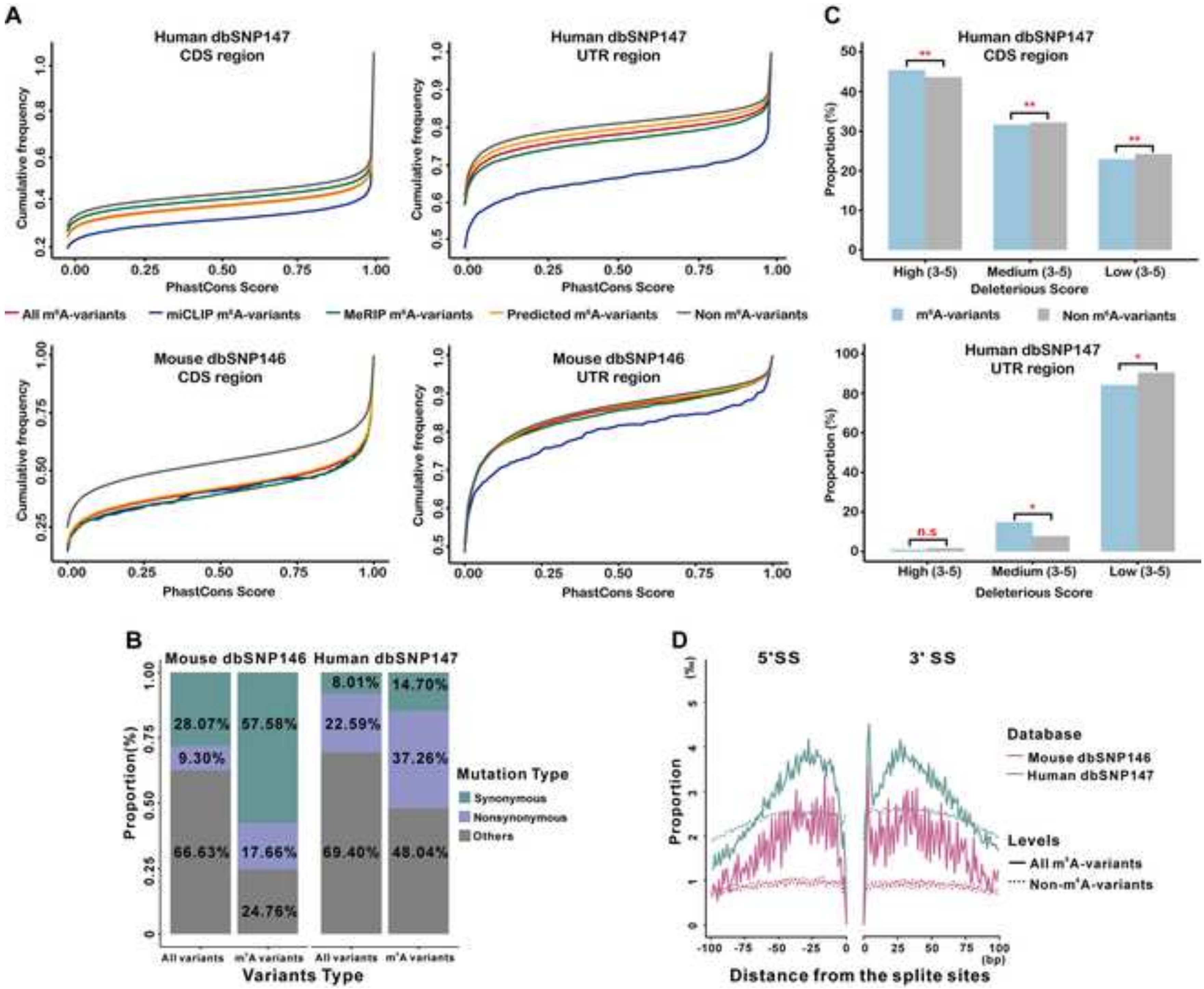

Table S1

Click here to access/download
Supplementary Material
Table S1.xlsx

Table S2

Click here to access/download
Supplementary Material
Table S2.xlsx

Table S3

Click here to access/download
Supplementary Material
Table S3.xlsx

Table S4

Click here to access/download
Supplementary Material
Table S4.xlsx

Supplementary figures

Click here to access/download
Supplementary Material
Supplementary figures.docx

Supplementary Data

Click here to access/download
Supplementary Material
Supplementary data.xlsx
