# Supplementary material for: m6ASNP: a tool for annotating genetic variants by m6A function
Source: Gigascience. 2018 Apr 2;7(5):giy035. doi: 10.1093/gigascience/giy035 (PMC6007280; doi:10.1093/gigascience/giy035)
Supplement: Supplemental material [file giy035_supp.zip › Supplementary figures.docx]

**SUPPLEMENTARY FIGURES**

**
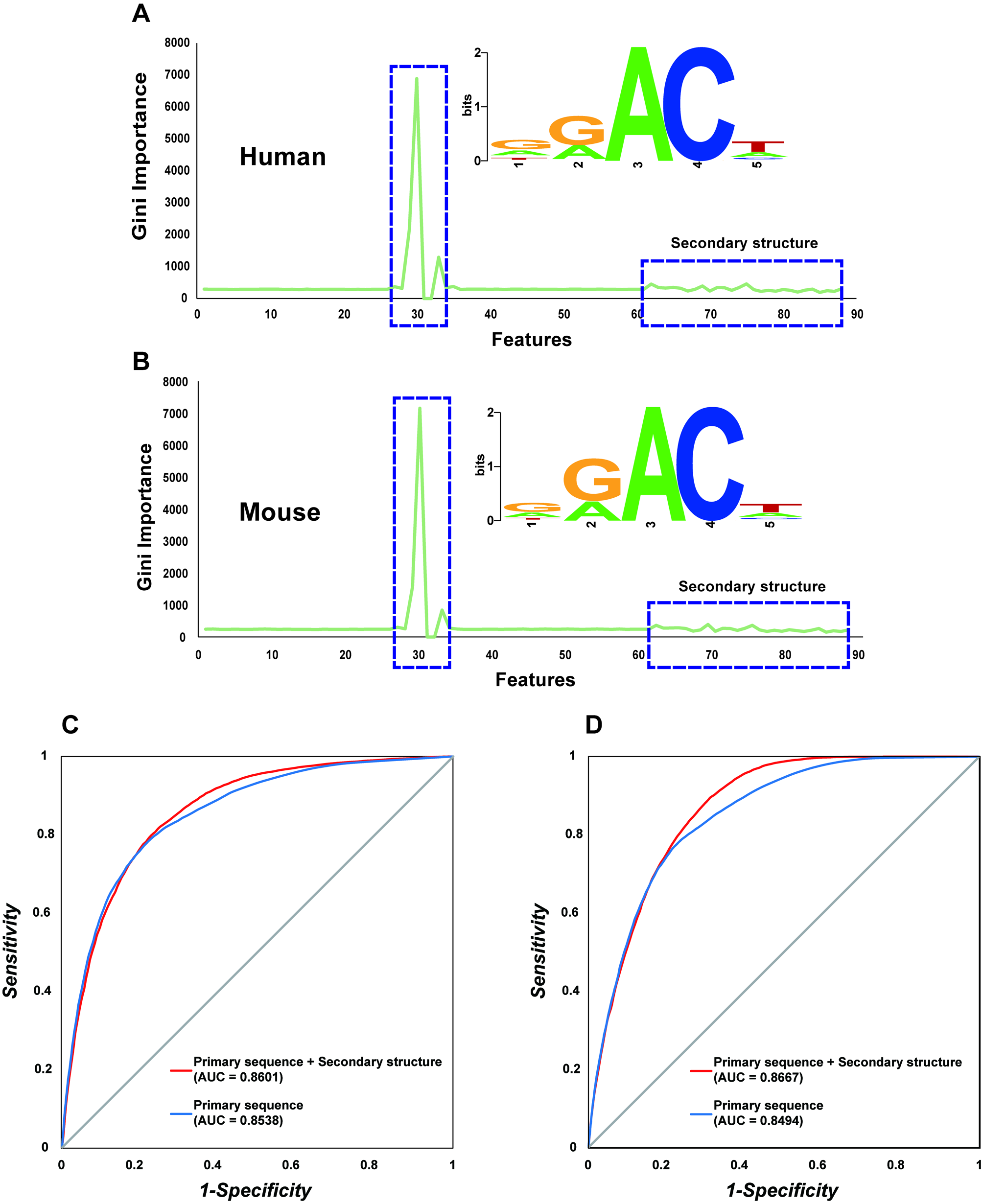
**

**Fig. S1 – The feature contribution of the human and mouse model.** Distribution plot of the feature’s Gini importance for both (A) human and (B) mouse model. The prediction capabilities of different combination of features for (C) human and (D) mouse model.


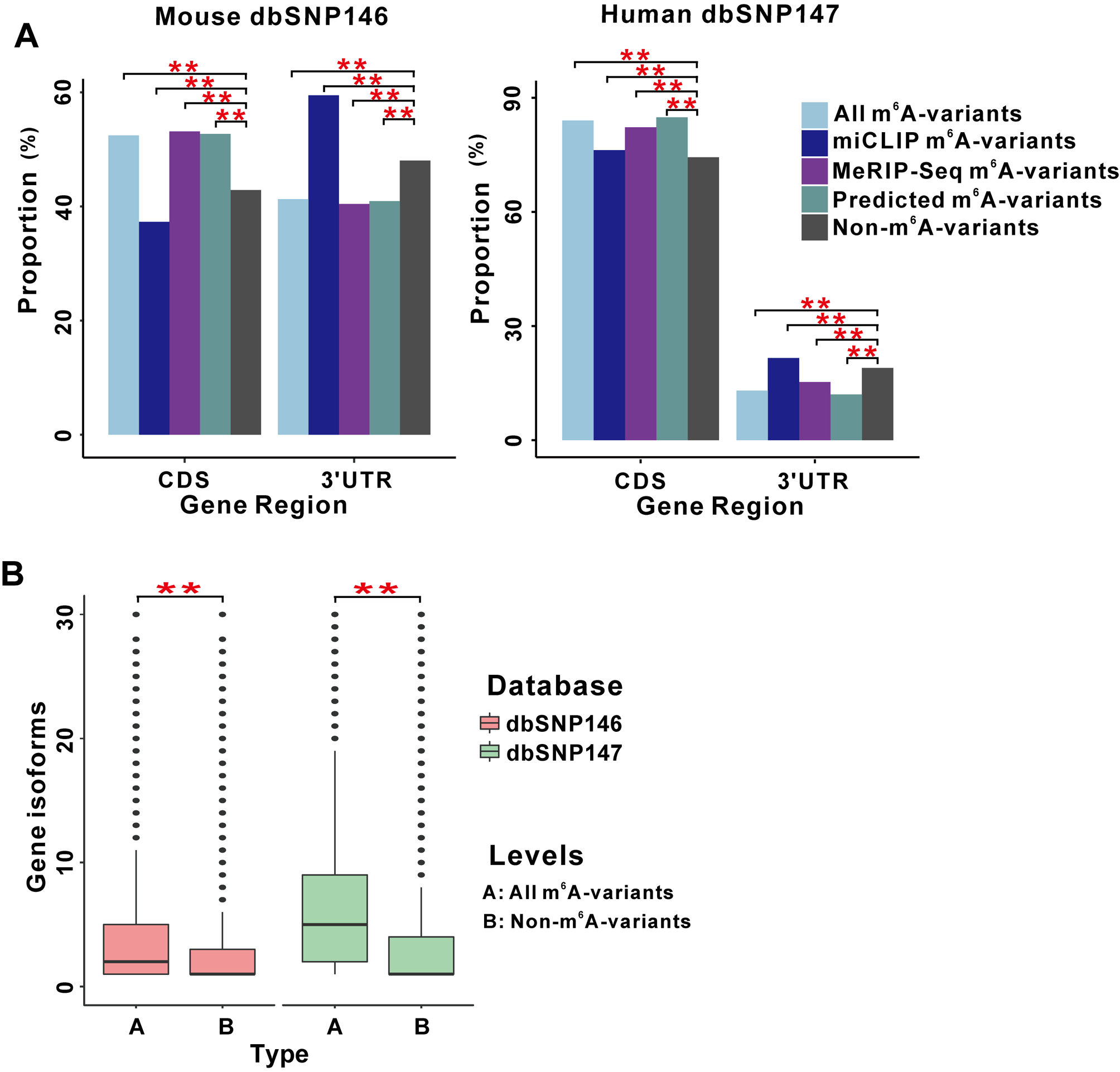


**Fig. S2 - A systematic comparison of the m^6^A-associated variants and non-m^6^A variants.** (A) Proportional distribution of the variants at different m^6^A confidence levels and non-m^6^A variants located in the CDS and 3’ UTR. A two-tailed test of population proportion was performed to assess significance. (B) Boxplots show the gene isoforms of the m^6^A-associated variants and non-m^6^A variants in different databases. One-sided Wilcoxon signed-rank test was performed to determine the significance. “**” indicates a significance level of P≤0.01, while “*” indicates P≤0.05.


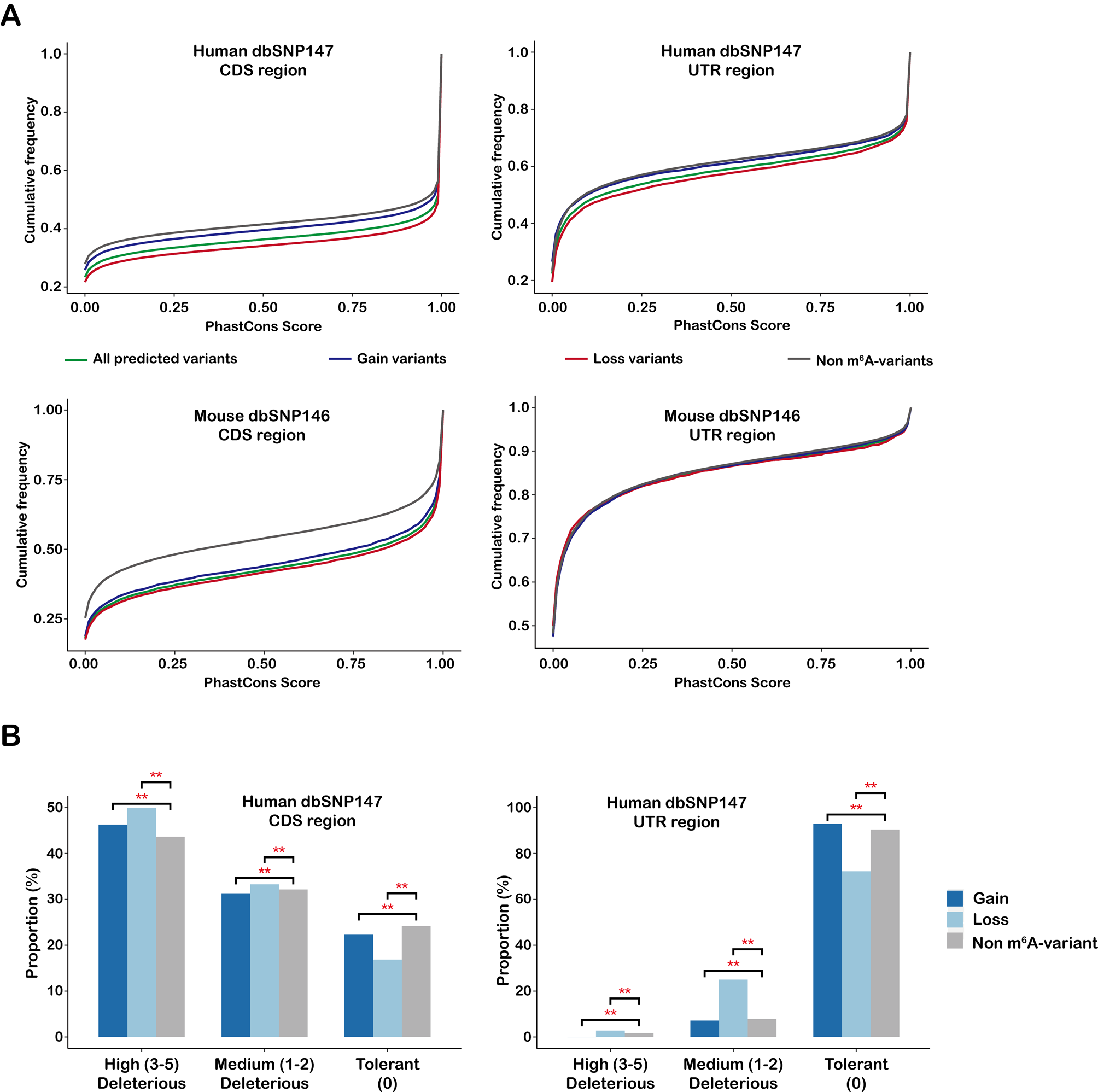


**Fig. S3 – The characteristics of m6A-associated variants predicted by m6ASNP.** (A) The conservation differences between functional gain and functional loss variants. (B) The comparison of mutation deleteriousness between functional gain and functional loss variants.


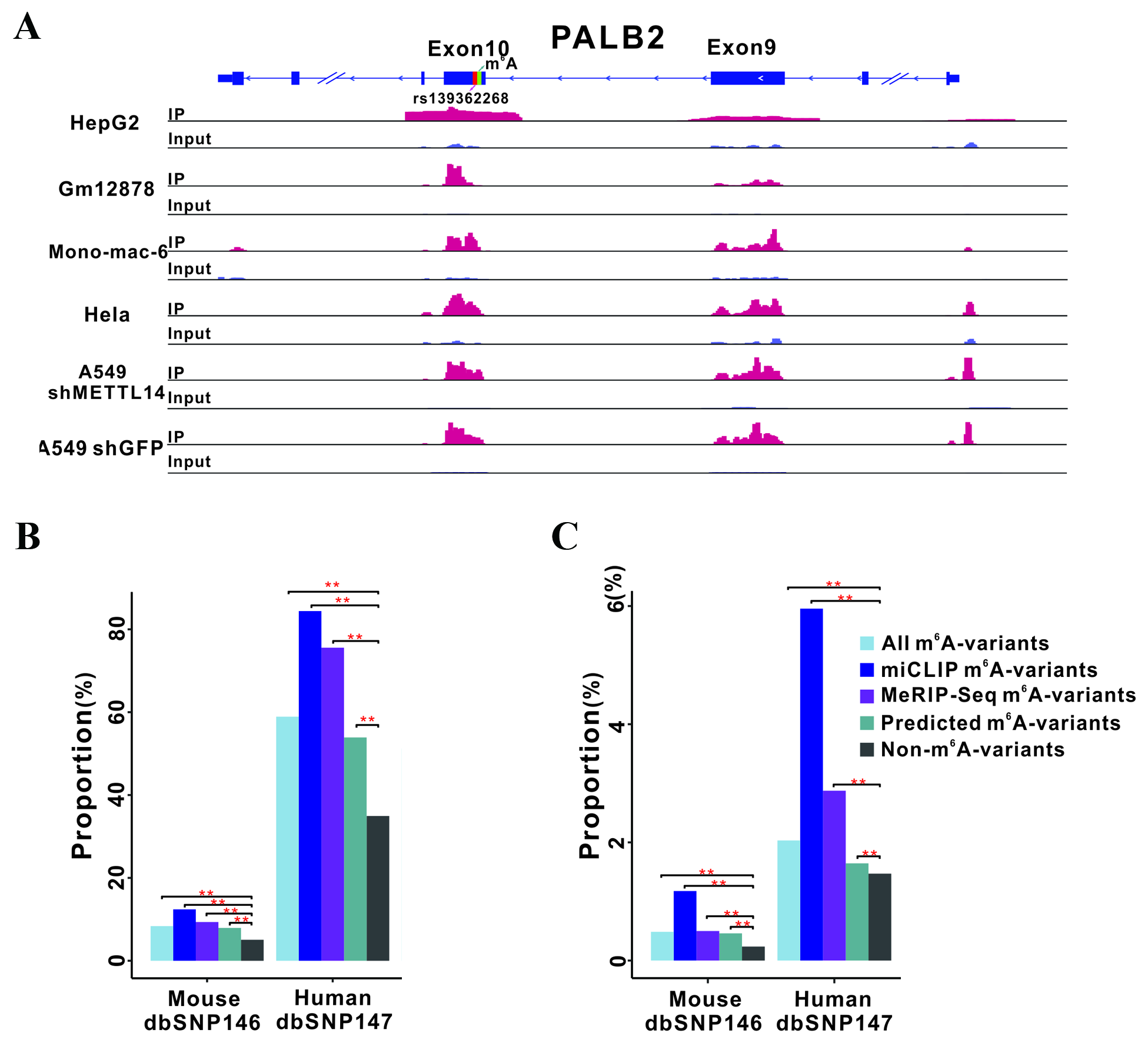


**Fig S4. Association analysis of m6A-associated variants.** (A) An example of m^6^A-associated variants in disease. The red rectangle in exon 10 represents a synonymous mutation in *PALB2,* i.e., rs139362268, while the green rectangle represents the m^6^A site. The 1 to 6 numbering indicates the different samples, followed by HepG2, GM12878, Momo-mac-6, HeLa, shMETTL14 in A549 and shGFP in A549. MeRIP-seq peak tracks of input, and the IP samples were scaled to the same level and colored in red and blue. (B-C) Proportional distribution of different levels of m^6^A-associated variants and non-m^6^A variants located within the RBP-binding regions and miRNA target regions. A two-tailed test of population proportion was performed to assess significance. “**” indicates a significance level of P≤0.01, while “*” indicates a significance level of P≤0.05.
