# Supplementary material for: m6ASNP: a tool for annotating genetic variants by m6A function
Source: Gigascience. 2018 Apr 2;7(5):giy035. doi: 10.1093/gigascience/giy035 (PMC6007280; doi:10.1093/gigascience/giy035)
Supplement: Response_to_Reviewer_Comments_Original_Submission.pdf [file giy035_response_to_reviewer_comments_original_submission.pdf]

Author's Response To Reviewer Comments

00000000-0000-(	BZaa2F5AbZR5(
E2A9317A	ZcO8NTc5At5k2t

Close

February 7, 2018

Editorial Office of GigaScience

Dear Dr. Scott Edmunds,

We are grateful for the prompt review of our manuscript, and helpful comments from two reviewers. According to the reviewer's comments, we have added more details for the methodologies and make more clear clarification on how to prepare the data set and construct the prediction tool. Besides, we also optimized the current web service to accept much larger input files in our server. To further support large-scale predictions, we have also provided a stand-alone program in our website. In addition, the software "m6ASNP" was approved by SciCrunch.org, and the RRID number is SCR_016048.

The manuscript was revised based on the reviewer's comments and all the revised texts were marked in red. Please find the point-by-point response below.

Thanks for your patience and coordination. I look forward to hearing from you.

Sincerely,

Jian Ren, PhD

Detailed Responses to Reviewers Comments

Reviewer 1#:

1. In introduction, the authors wrote that "Many studies have shown that abnormalities in post-transcriptional regulation are closely related to genetic diseases and complex diseases". However, only one reference was cited. The authors should cite more related references.

Response: Thank you for your suggestion. We have added two more latest references for this sentence.

2. Figure legends for Figure 1D is missing. Where are the mice data?

Response: We apologized for our carelessness. The construction processes of mouse data set were elaborated in the "Data collection" section. And evaluation results of the mouse model were also added in Figure 1.

In page 5, paragraph 1, added,
"Specifically, in Ke's paper, two tissue samples from mouse are also tested, from which we collected 8748 and 30078 N6-methyladenosines in liver and brain, respectively. We then combined these data … in human and 36,192 sites in mouse. For human model, we used… Similarly, for mouse model, 25334 m6A sites were preserved as positive training set, and another 10858 m6A sites were used as positive test set. From the human genome, we extracted… In the case of mouse genome, 1,519,570 adenine sites were extracted as negative training set and 625,600 adenine sites were constructed as negative test set."

In the legend of Figure 1, added,
"(D) The evaluation results of 4, 6, 8, 10-fold cross-validation in mouse model. (E) The performance comparison between m6ASNP and other state-of-art tools on the mouse test set."

3. How does deleterious score to be calculated? It should be described in detail in "Methods" section.

Response: The deleteriousness of a given variant is predicted using five state-of-art tools (SIFT, PolyPhen2 HVAR, PolyPhen2 HDIV, LRT and FATHMM). To produce a high confident representation of the variant deleteriousness, we defined an aggregate score ranging from 0 to 5 by counting the number of methods that consider an SNV to be deleterious. A higher value of the score indicates a higher probability of deleterious for a given variant. To make this clarification more clearly, we added the method detail as below.

In Page 13, paragraph 2, added,
"The deleteriousness of each variants was measured by integrating the prediction results from five pieces of software (SIFT, PolyPhen2 HVAR, PolyPhen2 HDIV, LRT and FATHMM). We defined an aggregate score by counting the number of the above methods that consider an SNV to be deleterious. A deleterious score of 0 means that the variant is predicted to be tolerated in both methods, while for a deleterious score of 5 means that the corresponding variant is predicted to be deleterious in all five predictors. As a result, the aggregate score may range from 0 to 5, and a higher score indicate a higher probability of deleterious."

4. In methods, the authors should provide more technical details (parameters, cutoffs, and workflow) on how to construct the model and how to perform association analyses. For example, how to determine miRNA targets? How to control false positives? What are the statistic tests for the association analyses?

Response: We have added more technical details in the method section. For the construction of random forest classifier, we have added the parameters used in training process. Also, detail procedures on how to derive and annotate the m6A-association variants were added. Specifically, in our association analyses, the miRNA targets were collected from previously published database such as starBase2 and CLIPdb. The statistical test for identifying significant m6A-association RBP regions and miRNA targets were a Monte-Carlo simulating method. The probabilities were calculated from an empirical distribution and the standard Benjamini-Hochberg method were applied to control the false positive rate. Besides, we also carefully checked the whole manuscript and ensured that all the statistical tests used in this study were clearly clarified. According to your suggestion, we revised our manuscript as listed below.

In page 5, paragraph 3, added,
"In order to identify the potential roles of m6A-associated variants in post-transcriptome regulation, the RBP binding sites from starBase2 and CLIPdb, the miRNA–RNA interactions from starBase2 and the canonical splice sites (GT-AG) from Ensembl annotations were collected. In addition, we also obtained a large number of disease-associated SNPs from different data sets (GWAS catalog, Johnson and O'Donnel, dbGAP, GAD and ClinVar) to perform disease-association analysis."

In page 11, paragraph 2, added,
"The random forest classifier for human and mouse were train separately on the above collected training set. The tree number was optimized as 500 and the features used for each splitting were set to 9. To assess the performance, we employed 4, 6, 8, 10-fold cross-validation on the training set. The additional test set was also applied in our study to evaluate the robustness. The sensitivity, specificity and Matthew's correlation coefficient were used to measure the predictor's performance."

In page 14, paragraph 3, added,
"This frequency may be regarded as an estimation of the probability that observing NB greater then NRBP in random condition. Next, the Benjamini-Hochberg method was applied to control the false positives."

5. I tested the webserver and found that the data size limit is < 500 KB. It would be nice if the authors provide a stand-alone version in case users have a large amount of data. Or they simply increase the file size limit.

Response: We have increased the file size limitation in our web server. Now, users can upload variant files up to 15 MB. Besides, to facilitate a more user-friendly experience, we also provided a stand-alone version in our website. The annotation files, genomic sequences as well as the complete documentation are available for download.

6. The webserver provides three thresholds to run the m6ASNP (high, medium and low). The authors should clarify what are these thresholds, and how to choose them.

Response: The high, medium and low threshold were selected from the evaluation results of 10-fold cross-validation by controlling the false positive rate at 0.05, 0.1 and 0.15, respectively. We added more detail descriptions in the main text to clarify this point. Besides, in the manuscript, we also made a suggestion on how to choose the appropriate threshold for the readers. The following is the revised text.

In page 6, paragraph 2, added,
“To balance the prediction accuracy, we selected three thresholds with high, medium and low stringencies for classification based on the evaluation result from 10-fold cross-validation. The high, medium and low thresholds were selected by controlling the false positive rate at 0.05, 0.1 and 0.15, respectively. Table 1 presented the detail performance under these three selected thresholds. In general, the high threshold provides the most stringent criterion and are usually used in large-scale prediction. The medium threshold is a balanced criterion and may be appropriate for most cases. The low threshold is the loosest criterion. When users expect to retain as much potential sites as possible, this threshold would be the best option.”

Reviewer #2:
Major comments:
1. It is not entirely clear to me how the test and training datasets are generated. While it is described how the human miCLIP datasets are generated, there is a lack of information on how the algorithm was trained for mouse (e.g. for the analyses in Figure 3). Or did the authors use the same random forest classifier that was trained with human m6A sites? If so, it should be stated explicitly. Similarly, different sets of m6A-associated variants (miCLIP, meRIP-Seq and predicted) are described in many of the analyses. Did the authors build another classifier using MeRIP-Seq training data? If so, as MeRIP seq does not provide single nucleotide resolution, how were the individual m6A sites inferred? The authors need to be more clear about this, especially as some of the later analyses show striking differences between the different annotations (e.g. 10 x the number of SNPs at MeRIP-sites compared to miCLIP sites [p. 6 lines 12-14] or differences in their conservation [Fig. 3A]).

Response: We have added more details on how the test and training dataset are constructed. In fact, we collected training and test datasets from two recently published miCLIP-seq data. The random forest models for human and mouse were trained and evaluated using the corresponding dataset, separately. The MeRIP-seq data were then collected for subsequent association analyses. We applied the random forest model trained from miCLIP-seq data to MeRIP-seq data and PA-m6A-seq to predict potential m6A sites in MeRIP-seq peaks and PA-m6A-seq sites. Therefore, we have three confidence levels of annotations: miCLIP-Seq and PA-m6A-seq sites (high confidence), predicted sites in MeRIP-Seq peaks (medium confidence) and other predicted sites (low confidence). To clarify this point more clearly, we added the following descriptions in the “Method” section.

In page 4, paragraph 4, added,
“To construct the prediction model, we first obtained the single-base-resolution m6A sites from two recently published miCLIP experiments. We collected 16,079 human m6A sites from Linder et al, and 43,155 human m6A sites from Ke et al. Specifically, in Ke’s paper, two tissue samples from mouse are also tested, from which we collected 8748 and 30078 N6-methyladenosines in liver and brain, respectively. We then combined these data sets to obtain a non-redundant data set that contains 55,548 sites in human and 36,192 sites in mouse. For human model, we used 35,871 non-redundant m6A sites as positive training set, and the rest 19,677 m6A sites were used as positive test set. Similarly, for mouse model, 25,334 m6A sites were preserved as positive training set, and another 10,858 m6A sites were used as positive test set. The negative data sets were generated according to the distribution of the positive sets. Because the majority of m6A sites conformed to a DRACH motif, we first defined the potential m6A sites as adenine sites that conform to the AC motif. Using the positive data sets as references, we extracted the non-methylated adenines that were followed by a cytosine in the same exon as the negative data set. From the human genome, we extracted 1,904,016 adenine sites as the negative training set, while the negative test set consisted of 1,286,588 adenine sites. In the case of mouse genome, 1,519,570 adenine sites were extracted as negative training set and 625,600 adenine sites were constructed as negative test set (Supplementary Data).”

In page 5, paragraph 2, added,
“To decipher the potential applications of m6ASNP, we further collected a complete set of genetic variants from dbSNP for human and mouse. The single-nucleotide variations (SNVs) within the exonic regions were preserved for subsequent analysis. Totally, 13,079,416 and 2,668,046 SNVs were collected in human and mouse, respectively. To investigate the potential role of these SNVs in reshaping the m6A event, m6A sites from two miCLIP-seq studies [42, 43], two PA-m6A-seq experiments [44] and 244 MeRIP-seq samples were integrated. Using m6ASNP, we further predicted the potential m6A-associated variants from the above data set. Besides, a transcriptome-wide prediction was also performed. Overall, 311,706 and 40,308 m6A-associated variants were obtained from human and mouse, respectively.”

In page 12, paragraph 2, added,
“Based on miCLIP-seq, PA-m6A-seq and MeRIP-seq data, we then combined them with the SNV data from dbSNP and performed m6A-association prediction using m6ASNP. Following the same procedure

proposed in our previously published work [60], we constructed three confidence levels of annotations of m6A-associated variants for subsequent analysis.
The first annotation was the high confidence level data that contained the m6A-associated variants derived from miCLIP-seq and PA-m6A-seq experiments. Notably, the PA-m6A-seq can only detect m6A signal in a resolution of ~23nt, therefore, to obtain precise modification sites, we scanned through all the peak regions and extracted adenosine sites that conformed to DRACH motif as final m6A sites. On this basis, we retained the variants that located nearby the m6A sites as the m6A-associated variants.
The second annotation was the medium confidence level data. We first downloaded all the published MeRIP-seq data from GEO database. According to the standard analysis pipeline for MeRIP-seq data, we applied MACS2, MeTPeak and Meyer's method to identify the m6A peaks in each study separately. MSPC was then applied to construct consensus peaks from the above three methods. In those consensus peaks, we then applied m6ASNP to predict m6A-associated variants that significantly change the DRACH motif.
The third annotation was the low confidence level data, where we used the whole transcriptome sequences for prediction. With a high threshold, m6ASNP will predict the potential m6A-associated variants from all collected genetic variants.
In summary, we had constructed 13,703 high confidence level, 54,222 medium confidence level and 243,880 low confidence level of m6A-associated variants for human. Another 935 high confidence level, 9,404 medium confidence level and 17,739 low confidence level data were also constructed for mouse.”

2. The authors state that "m6A-associated variants were enriched in protein-coding genes" and "significantly concentrated in CDS and 3'UTR" [p.6 lines 20-21]. It is not clear from the referenced Figure S1A or the methods section how this was calculated.

Response: We have added a detailed protocol about the above analysis in the method section.

In Page 13, paragraph 2, added,
“All the identified m6A-association variants were annotated by the transcript structure, including the CDS, 3’ UTR, 5’ UTR, start codon and stop codon etc. For the annotation of non-coding RNA, the DASHR, miRBase(version 21), GtRNAdb and piRNABank were used. To test whether the m6A-association variants were more preferentially distributed in specific transcript structures, we calculated the proportion of variants that located in a given transcript structure. In order to avoid bias, only the variants that were annotated in mRNA were used, and the proportion in 5’-UTR, CDS and 3’-UTR were calculated. A two-tailed proportion test was then adopted to compare the proportion difference between m6A-association variants and non-m6A variants.”

3. As m6A sites themselves are enriched in the CDS and 3'UTR, this has to be taken into account in order to make such a statement. This becomes even more important, as the proportions of variants in the different regions in Fig. S1 do not add up. This suggests that the authors were not restricting their analysis to variants that map to mRNA. This is an issue, as most of the non-m6A-variants seem to be located outside of mRNA transcripts, whereas the m6A-variants naturally occur almost exclusively in mRNAs.

Response: The m6A-association variants observed in our analysis were mainly enriched in the CDS and 3’ UTR, which is in line with the fact that m6A sites were more likely to occur in CDS and 5’-UTR regions. Our original intention was to illustrate that the identified m6A-association variants agreed well with the known characteristics of m6A sites, indicating the robustness of our predictor. Therefore, we have modified our statement and made this point more clearly. As for Fig. S1, we have reanalyzed the data and restricted the test to variants that map to mRNA. The original Fig. S1 was changed to Fig. S2. To show entire results, the 5’-UTR is added (Table S1). Now proportions in different regions should be summed up to 1.

4. Therefore, without proper filtering two very different sets of variants are compared in the downstream analyses. This can be seen in Figure 3A, where the conservation of m6A-associated variants is analyzed. If most of the non-m6A-variants are located in intergenic regions, it is totally expected that they are less conserved. Therefore, in order to test whether there is indeed a higher selective pressure on the m6A-variants as compared to the non-m6A-variants, they have to be compared to the non-m6A-variants in mRNAs (preferably even in the same exons).

Response: In fact, the analyses of m6A-association variants were performed in exonic regions. We have already filtered the intronic and intergenic regions before the analysis in Fig. 3A. To clarify this point more clearly, we added some descriptions in the method section. Furthermore, we also tested the conservative and deleterious differences between m6A-association variants and non-m6A variants in the same exons, the result of which were shown in the modified Fig. 3. Interestingly, the statistical test was still significant even under this strict condition. We have revised our manuscript as shown below.

In page 13, paragraph 2, added,
“To avoid any bias, we only preserved those variants located in mRNA for analysis, and compared the conservative and deleterious differences between m6A-association variants and non-m6A variants in the same exon.”

Minor comments:
1. Most analyses would benefit from distinguishing "functional gain" from "functional loss" variants, as they are expected to behave differently. For example, while there might be selective pressure on existing m6A sites (which are affected by "functional loss" variants), this is expected to be less at "functional gain" variants.

Response: This is a very good point. To investigate the different behavior of functional gain or functional loss m6A-associated variants, we divided the predicted variants into two corresponding groups and compared their conservation and deleteriousness in Fig S3. As expected, the loss of existing m6A sites may undergo a stronger selective pressure comparing to the gain mutations of m6A sites, and this phenomenon was more pronounced in human. Interestingly, when comparing their deleteriousness, we also found that functional loss variants may have a far greater impact on the function of a given transcript. Taken together we can suggest that the disruption of existing m6A sites may be an important mechanism for the imbalance of cell function, and which can be served as a potential targets for future therapeutic research.

In page 7, paragraph 2, added,
“To further dissert the functional role of m6A-associated variants, we divided the predicted m6A-associated variants into two groups, that is the functional gain and functional loss variants. The same conservation analysis was performed on these groups and the results were compared to non-m6A variants (Fig. S3A). Strikingly, the functional loss variants were found to be more conservative comparing to the gain variants, and which suggesting that the loss of existing m6A sites may undergo stronger selective pressure than the gain mutations on potential adenylate sites. Moreover, m6A-associated variants were also predicted to be more deleterious than non-m6A variants (Fig. 3C, two-tailed population test). Again, the functional loss variants appeared to have a higher deleteriousness comparing to the functional gain variants (Fig S3B). Taken together, we can conclude that m6A-associated variants, especially the functional loss variants, may have important roles and could be driven by positive selection in mammalian genomes.”

2. What is the contribution of structural features to the model? From the test data available on the server, it seems that most called m6A-associated variants directly affect the m6A motif.

Response: To address this issue, we have retrained our model for both human and mouse data set, and evaluated their feature contributions using Gini importance in Figure S1A. Indeed, for m6A sites prediction, the primary sequence is the most important type of features. Especially in the m6A motif region, the flanking nucleotides contributed most significantly for classification of potential m6A sites. However, we also observed slight contribution of secondary structure in both human and mouse model. Although the weak contribution, we expected that the secondary structure may bring extra information for prediction and improve the accuracy and the robustness of our models. These can be seen in Figure S1B. As the addition of secondary structure, the performance for both human and mouse model are promoted. Therefore, in our final models, the primary sequence feature and the secondary structure feature were combined. To clarify this point more clearly, we added the following statements in the main text.

In page 6, paragraph 1, added,
“In order to evaluate the contribution of different encoding features, we first computed the mean decrease of Gini impurity (also known as Gini importance) for the human and mouse model. The distribution plot of Gini importance in different features showed that the primary sequence was the most effective feature for predicting potential m6A sites. Nucleotides in the DRACH motif around the N6-methyladenosine were dominated for classification (Fig S1A). However, secondary structures were still observed to contribute the prediction of m6A sites. Further evaluation on the prediction capability of primary sequence and secondary structure indicated that the addition of structural features to the sequence features can improve the accuracy and robustness of both models (Fig S1B). Therefore, in the final model of both human and mouse, we combined those features together to obtain a better performance.”

3. In the introduction, the link between FTO SNVs and obesity/diabetes [p. 4 lines 9-10] needs to be toned down, as the FTO intronic variants likely act via changing the expression of neighboring genes and not FTO itself (Smemo et al., 2014; 10.1038/nature13138).

Response: Thanks for your comments. We have revised this sentence as your suggestion.

In page 4, paragraph 1, revised,
“The mutation on FTO, an m6A demethylase, can change the expression level of neighboring genes and therefore leading to obesity and type 2 diabetes [27].”

4. The analysis of the RBP binding sites should be moved from the discussion section to the results section.

Response: We have moved the analysis of RBP-binding sites, miRNA targets and related diseases from discussion section to the result section. Correspondingly, we also added some conclusions in the original text.

In page 10, paragraph 2, added,
“Using m6ASNP, we performed further functional analysis on m6A-associated variants. By integrating data set regarding RBP-binding regions, miRNA-targets and splicing sites, m6ASNP can help to reveal the potential relationship among variants, m6A modification and other post-transcriptional regulation. Also, the disease-association analysis had identified more than 2,000 disease-related variants that may be linked with alterations of m6A modification. This finding further proved that m6ASNP is a promising tool for studying the potential role of m6A variants in clinical investigation.”

5. There are a lot of typos throughout the manuscript, even in the figures (Fig. 3B).

Response: We are sorry for the typos. We have gone through our manuscript again and corrected all the typos in the main text.

Close
