# Supplementary material for: m6ASNP: a tool for annotating genetic variants by m6A function
Source: Gigascience. 2018 Apr 2;7(5):giy035. doi: 10.1093/gigascience/giy035 (PMC6007280; doi:10.1093/gigascience/giy035)
Supplement: Response_to_Reviewer_Comments_Revision_1.pdf [file giy035_response_to_reviewer_comments_revision_1.pdf]

Author's Response To Reviewer Comments

00000000-0000-(	soVn6yr9fDTE+G
E2A9317A	cv71/m89MGM8iE

Close

March 10, 2018

Editorial Office of GigaScience

Dear Dr. Scott Edmunds,

We are grateful for the prompt review of our manuscript. According to reviewer #2's comments, we have divided the conservation and deleteriousness analysis into UTR and CDS groups. The corresponding figures and texts were revised accordingly. Besides, the whole text was carefully gone over again, and we have tried our best to correct all typos.

The manuscript was revised based on the reviewer's comments and all the revised texts were marked in red. Please find the point-by-point response below.

Thanks for your patience and coordination. I look forward to hearing from you.

Sincerely,

Jian Ren, PhD

Detailed Responses to Reviewers Comments

Reviewer 2#:

1. On p. 12 line 40, it is stated that "MSPC was then applied to construct consensus peaks". An explanation for the abbreviation MSPC is not given and it is thus unclear how the consensus of MeRIP-Seq peaks was determined.

Response: MSCP is a tool designed by Jalili et al, and it is originally used for combining overlapped peaks from different ChIP-seq replicates. Generally speaking, if a given region is identified as enriched in most of the adopted methods, it is more likely to be a true modification signal in MeRIP-seq experiment. Therefore, to obtain reliable m6A peaks, here we applied MSCP to pick up enriched regions that identified by most of the current peak calling algorithms. To make this point more clearly, we have revised this sentence as shown below and added a citation for this tool.

In page 3, paragraph 4, revised,
"Generally speaking, if a given region is identified as enriched in most of the adopted methods, it is more likely to be a true modification signal in MeRIP-seq experiment. Therefore, to obtain reliable m6A peaks, a tool called MSPC was then applied to construct consensus peaks from the above three methods."

2. However, I still think that the claim that "m6A-associated variants [...] could be driven by positive selection" (p. 8 line 17) is not fully supported by the current data. The reason is that there is still significant positional bias in the compared groups (m6A variants vs. non-m6A variants). While I agree that the enrichment of m6A-variants in CDS (and 3'UTR) points towards their validity of being m6A sites, this makes a comparison with the non-m6A variants difficult. The problem is identical to the one arising from comparing variants in genic and intergenic regions: The two regions compared are under different selective pressure. The same holds true also for coding sequence vs untranslated regions, as the latter are typically less conserved. Thus, the enrichment of m6A-variants in the CDS (see the supplementary table 1) leads to an oversampling of this region that predicts that these variants are on average more conserved and more deleterious. Therefore, as long as the oversampling of CDS is not taken into account, it is difficult to conclude that m6A-variants are indeed more conserved than their

non-m6A counterparts. One way to control for this might be to analyze CDS and UTRs separately or to subsample the sites so that each set has a similar proportion of these regions.

Response: We are grateful for your careful review. We agreed that the selective pressures are different in the protein-coding sequence and the untranslated region. And therefore, there may be bias in our results. To eliminate such bias, we have reanalyzed our data and restricted the comparisons in only CDS or UTR region. Figure 3 and Figure S3 were modified as you suggested. Specifically, the conservation scores still have significant differences between m6A-associated variants and non-m6A variants in both CDS and UTR region. The conservation differences in human data were larger than that in mouse data. Similarly, the deleteriousness also confirmed to the above characteristics. In most cases, the deleteriousness of m6A-associated variants are higher than that of non-m6A variants. According to these new results, the corresponding texts in the manuscript were revised as listed below.

In page 7, paragraph 2, revised,
"Interestingly, in both CDS and UTR region, m6A-associated variants were more conserved than non-m6A variants (Fig. 3A). For those conserved m6A-associated variants, a significant portion was synonymous compared to all conserved variants (Fig. 3B, $p < 0.0001$, hypergeometric test). To further explain the functional role of m6A-associated variants, we divided the predicted m6A-associated variants into two groups: the functional gain and functional loss variants. The conservation analysis was performed on these two groups and the results were compared to non-m6A variants in both CDS and UTR region (Fig. S3A). Strikingly, in most cases, the functional loss variants were found to be more conservative comparing to the gain variants, suggesting that the loss of existing m6A sites may undergo stronger selective pressure than the gain mutations on potential adenylate sites. Moreover, m6A-associated variants were predicted to be more deleterious than non-m6A variants in both the CDS and UTR region (Fig. 3C, two-tailed population test). Again, for the predicted data, the functional loss variants appeared to have a higher deleteriousness comparing to the functional gain variants and the non-m6A variants (Fig S3B)."

In page 13, paragraph 1, added,
"As the selective pressures were quite different in protein-coding sequences and untranslated regions, the above comparison was carried out separately for the CDS and UTR regions."

3. The sentence was now changed to "The mutation on FTO, an m6A demethylase, can change the expression level of neighboring genes and therefore leading to obesity and type 2 diabetes [27]." (p4. line 9). This misses the point that mutations in FTO have not been linked to obesity and type 2 diabetes. The loss of function mutations in the protein coding region that interfere with the demethylase activity of FTO lead to a severe developmental phenotype (as the authors correctly state on p4. lines 11-17). The link of the FTO locus with obesity comes from intronic SNVs, which are reported to regulate expression of neighboring genes rather than FTO activity. Thus, a direct link between FTO activity and obesity/diabetes is still elusive.

Response: We appreciate for your careful review. To avoid any incorrect presentation or misunderstanding, we have deleted this sentence from the manuscript.

4. There are still some typos (e.g. p7 line 58 "To further dissert the functional role" or p. 8 line 33 "have revealed many diseases related variants").

Response: Sorry again for the typos! We have corrected the above typos.

Close
