# Supplementary material for: m6ASNP: a tool for annotating genetic variants by m6A function
Source: Gigascience. 2018 Apr 2;7(5):giy035. doi: 10.1093/gigascience/giy035 (PMC6007280; doi:10.1093/gigascience/giy035)
Supplement: Response_to_Reviewer_Comments_Revision_2.pdf [file giy035_response_to_reviewer_comments_revision_2.pdf]

Author's Response To Reviewer Comments

Dear Editors,

We have rearranged the orders for the supplementary files.

Best,

Zhixiang
