# Supplementary material for: m6ASNP: a tool for annotating genetic variants by m6A function
Source: Gigascience. 2018 Apr 2;7(5):giy035. doi: 10.1093/gigascience/giy035 (PMC6007280; doi:10.1093/gigascience/giy035)
Supplement: Reviewer_1_Report_(Original_Submission) -- Yun-Gui Yang — 1/3/2018 Reviewed [file giy035_reviewer_1_report_(original_submission).pdf]

Reviewer Report

Title: m6ASNP: a tool for annotating genetic variants by m6A function

Version: Original Submission **Date:** 1/3/2018

Reviewer name: Yun-Gui Yang

Reviewer Comments to Author:

The manuscript "m6ASNP: a tool for annotating genetic variants by m6A function" by Jiang et al. described a new web server for the identification of genetic variants associated with m6A modification sites. N6-methyladencosine (m6A) is the most abundant modification in mRNA molecules and has been known to play important roles in post-translational regulatory mechanism. Till now, there is still lack of tools to evaluate whether mutations affect the methylation status of m6A sites nearby. The authors developed an m6A prediction model for predicting whether a variant alters the methylation status of m6A sites at the nearby AC motif. Moreover, to facilitate the use of their tool, the authors developed a web-based service called m6ASNP to implement this prediction tool, which may benefit the understanding of the effects of variants, especially those synonymous variants. The manuscript is clearly written and the web server is well designed. I would like to recommend it to be accepted if the authors could address the following concerns.1. In introduction, the authors wrote that "Many studies have shown that abnormalities in post-transcriptional regulation are closely related to genetic diseases and complex diseases". However, only one reference was cited. The authors should cite more related references.2. Figure legends for Figure 1D is missing. Where are the mice data? 3. How does deleterious score to be calculated? It should be described in detail in "Methods" section.4. In methods, the authors should provide more technical details (parameters, cutoffs, and workflow) on how to construct the model and how to perform association analyses. For example, how to determine miRNA targets? How to control false positives? What are the statistic tests for the association analyses?5. I tested the webserver and found that the data size limit is < 500 KB. It would be nice if the authors provide a stand-alone version in case users have a large amount of data. Or they simply increase the file size limit.6. The webserver provides three thresholds to run the m6ASNP (high, medium and low). The authors should clarify what are these thresholds, and how to choose them.

Level of Interest

Please indicate how interesting you found the manuscript: An article of importance in its field

Quality of Written English

Please indicate the quality of language in the manuscript: Acceptable

Declaration of Competing Interests

Please complete a declaration of competing interests, considering the following questions:

- Have you in the past five years received reimbursements, fees, funding, or salary from an organisation that may in any way gain or lose financially from the publication of this manuscript, either now or in the future?

- Do you hold any stocks or shares in an organisation that may in any way gain or lose financially from the publication of this manuscript, either now or in the future?
- Do you hold or are you currently applying for any patents relating to the content of the manuscript?
- Have you received reimbursements, fees, funding, or salary from an organization that holds or has applied for patents relating to the content of the manuscript?
- Do you have any other financial competing interests?
- Do you have any non-financial competing interests in relation to this paper?

If you can answer no to all of the above, write 'I declare that I have no competing interests' below. If your reply is yes to any, please give details below.

I declare that I have no competing interests

I agree to the open peer review policy of the journal. I understand that my name will be included on my report to the authors and, if the manuscript is accepted for publication, my named report including any attachments I upload will be posted on the website along with the authors' responses. I agree for my report to be made available under an Open Access Creative Commons CC-BY license (http://creativecommons.org/licenses/by/4.0/). I understand that any comments which I do not wish to be included in my named report can be included as confidential comments to the editors, which will not be published.

I agree to the open peer review policy of the journal

To further support our reviewers, we have joined with Publons, where you can gain additional credit to further highlight your hard work (see: https://publons.com/journal/530/gigascience). On publication of this paper, your review will be automatically added to Publons, you can then choose whether or not to claim your Publons credit. I understand this statement.

Yes
