# Supplementary material for: m6ASNP: a tool for annotating genetic variants by m6A function
Source: Gigascience. 2018 Apr 2;7(5):giy035. doi: 10.1093/gigascience/giy035 (PMC6007280; doi:10.1093/gigascience/giy035)
Supplement: Reviewer_2_Report_(Original_Submission) -- Bastian Linder — 18 Jan 2018 Reviewed [file giy035_reviewer_2_report_(original_submission).pdf]

Reviewer Report

Title: m6ASNP: a tool for annotating genetic variants by m6A function

Version: Original Submission **Date:** 18 Jan 2018

Reviewer name: Bastian Linder

Reviewer Comments to Author:

In this manuscript, Jiang, Xie and He et al. describe an approach to annotate single nucleotide polymorphisms / variants (SNPs / SNVs) with information about the methylation status of nearby RNA modification sites. They focus on N6-methyladenosine (m6A), an RNA modification that has gained a lot of attention recently, as it has been proposed to control a variety of RNA metabolic activities and was linked to a number of diseases, including cancer.They develop a random forest classifier to determine whether putative m6A sites are methylated. They then use this algorithm to classify SNPs into those that are predicted to create novel m6A sites ("functional gain") or abrogate existing m6A sites ("functional loss"). This method of annotating clinically interesting variants with m6A information will be valuable, as m6A is involved in regulating mRNA stability and thus has the potential to control gene expression. Therefore, the method presented here would be a highly valuable tool for the scientific community, especially for genetic association studies. However, the manuscript lacks some critical information about how the algorithm was developed. Also, the exemplary annotation of dbSNP of ClinVar and dbSNP data with functional m6A data has some issues.If the authors can adequately address these issues, the paper would be suitable for publication in GigaScience.Major comments:1.) It is not entirely clear to me how the test and training datasets are generated. While it is described how the human miCLIP datasets are generated, there is a lack of information on how the algorithm was trained for mouse (e.g. for the analyses in Figure 3). Or did the authors use the same random forest classifier that was trained with human m6A sites? If so, it should be stated explicitly. Similarly, different sets of m6A-associated variants (miCLIP, meRIP-Seq and predicted) are described in many of the analyses. Did the authors build another classifier using MeRIP-Seq training data? If so, as MeRIP seq does not provide single nucleotide resolution, how were the individual m6A sites inferred? The authors need to be more clear about this, especially as some of the later analyses show striking differences between the different annotations (e.g. 10 x the number of SNPs at MeRIP-sites compared to miCLIP sites [p. 6 lines 12-14] or differences in their conservation [Fig. 3A]).2.) The authors state that "m6A-associated variants were enriched in protein-coding genes" and "significantly concentrated in CDS and 3'UTR" [p.6 lines 20-21]. It is not clear from the referenced Figure S1A or the methods section how this was calculated. As m6A sites themselves are enriched in the CDS and 3'UTR, this has to be taken into account in order to make such a statement. This becomes even more important, as the proportions of variants in the different regions in Fig. S1 do not add up. This suggests that the authors were not restricting their analysis to variants that map to mRNA. This is an issue, as most of the non-m6A-variants seem to be located outside of mRNA transcripts, whereas the m6A-variants naturally occur almost exclusively in mRNAs. Therefore, without proper filtering two very different sets of variants are compared in the downstream analyses.This can be seen in Figure 3A, where the conservation of m6A-associated variants is analyzed. If most of the non-m6A-variants are located in intergenic regions, it is totally expected that they are less conserved. Therefore, in order to test whether there is indeed a higher selective pressure on the m6A-variants as compared to the non-m6A-variants, they have to be compared to the non-m6A-variants in mRNAs (preferably even in the same exons). It would be interesting to see whether the finding that m6A-associated variants are more conserved remains valid, especially in light of recent findings suggesting that there is little selective pressure on individual m6A sites (Liu and Zhang, Mol Biol Evol. 2018, adv. online doi:10.1093/molbev/msx320).Minor comments:1.) Most analyses would benefit from distinguishing "functional gain" from "functional loss" variants, as they are expected to behave differently. For example, while there might be selective pressure on existing m6A sites (which are affected by "functional loss" variants), this is expected to be less at "functional gain" variants.2.) What is the contribution of structural features to the model? From the test data available on the server, it seems that most called m6A-associated

variants directly affect the m6A motif.3.) In the introduction, the link between FTO SNVs and obesity/diabetes [p. 4 lines 9-10] needs to be toned down, as the FTO intronic variants likely act via changing the expression of neighboring genes and not FTO itself (Smemo et al., 2014; 10.1038/nature13138). 4.) The analysis of the RBP binding sites should be moved from the discussion section to the results section.5.) There are a lot of typos throughout the manuscript, even in the figures (Fig. 3B).

Level of Interest

Please indicate how interesting you found the manuscript: An article of importance in its field

Quality of Written English

Please indicate the quality of language in the manuscript: Needs some language corrections before being published

Declaration of Competing Interests

Please complete a declaration of competing interests, considering the following questions:

- Have you in the past five years received reimbursements, fees, funding, or salary from an organisation that may in any way gain or lose financially from the publication of this manuscript, either now or in the future?
- Do you hold any stocks or shares in an organisation that may in any way gain or lose financially from the publication of this manuscript, either now or in the future?
- Do you hold or are you currently applying for any patents relating to the content of the manuscript?
- Have you received reimbursements, fees, funding, or salary from an organization that holds or has applied for patents relating to the content of the manuscript?
- Do you have any other financial competing interests?
- Do you have any non-financial competing interests in relation to this paper?

If you can answer no to all of the above, write 'I declare that I have no competing interests' below. If your reply is yes to any, please give details below.

I declare that I have no competing interests

I agree to the open peer review policy of the journal. I understand that my name will be included on my report to the authors and, if the manuscript is accepted for publication, my named report including any attachments I upload will be posted on the website along with the authors' responses. I agree for my report to be made available under an Open Access Creative Commons CC-BY license (http://creativecommons.org/licenses/by/4.0/). I understand that any comments which I do not wish to

be included in my named report can be included as confidential comments to the editors, which will not be published.

I agree to the open peer review policy of the journal

To further support our reviewers, we have joined with Publons, where you can gain additional credit to further highlight your hard work (see: https://publons.com/journal/530/gigascience). On publication of this paper, your review will be automatically added to Publons, you can then choose whether or not to claim your Publons credit. I understand this statement.

Yes
