# Supplementary material for: m6ASNP: a tool for annotating genetic variants by m6A function
Source: Gigascience. 2018 Apr 2;7(5):giy035. doi: 10.1093/gigascience/giy035 (PMC6007280; doi:10.1093/gigascience/giy035)
Supplement: Reviewer_2_Report_(Revision_1) -- Bastian Linder — 2/22/2018 Reviewed [file giy035_reviewer_2_report_(revision_1).pdf]

Reviewer Report

Title: m6ASNP: a tool for annotating genetic variants by m6A function

Version: Revision 1 **Date:** 2/22/2018

Reviewer name: Bastian Linder

Reviewer Comments to Author:

Reviewer #2:Major comments:1. It is not entirely clear to me how the test and training datasets are generated. While it is described how the human miCLIP datasets are generated, there is a lack of information on how the algorithm was trained for mouse (e.g. for the analyses in Figure 3). Or did the authors use the same random forest classifier that was trained with human m6A sites? If so, it should be stated explicitly. Similarly, different sets of m6A-associated variants (miCLIP, meRIP-Seq and predicted) are described in many of the analyses. Did the authors build another classifier using MeRIP-Seq training data? If so, as MeRIP seq does not provide single nucleotide resolution, how were the individual m6A sites inferred? The authors need to be more clear about this, especially as some of the later analyses show striking differences between the different annotations (e.g. 10 x the number of SNPs at MeRIP-sites compared to miCLIP sites [p. 6 lines 12-14] or differences in their conservation [Fig. 3A]).Response: We have added more details on how the test and training dataset are constructed. In fact, we collected training and test datasets from two recently published miCLIP-seq data. The random forest models for human and mouse were trained and evaluated using the corresponding dataset, separately. The MeRIP-seq data were then collected for subsequent association analyses. We applied the random forest model trained from miCLIP-seq data to MeRIP-seq data and PA-m6A-seq to predict potential m6A sites in MeRIP-seq peaks and PA-m6A-seq sites. Therefore, we have three confidence levels of annotations: miCLIP-Seq and PA-m6A-seq sites (high confidence), predicted sites in MeRIP-Seq peaks (medium confidence) and other predicted sites (low confidence). To clarify this point more clearly, we added the following descriptions in the "Method" section.Reviewer's response to 1.):The description of how the m6A variants were generated has now improved. Given that most of the information in the random forest classifier seems to come from changes to the DRACH consensus motif, this method is somewhat similar to picking a variant that changes a DRACH (either in m6A-enriched peaks from MeRIP-Seq experiments or in the whole transcriptome). I am not sure whether this approach is accurate for DRACH motifs that have not been directly demonstrated to be methylated. However, the different confidence levels are clearly stated in the manuscript so the reader can assess this and the revised version satisfies my concerns. One minor point: On p. 12 line 40, it is stated that "MSPC was then applied to construct consensus peaks". An explanation for the abbreviation MSPC is not given and it is thus unclear how the consensus of MeRIP-Seq peaks was determined.2. The authors state that "m6A-associated variants were enriched in protein-coding genes" and "significantly concentrated in CDS and 3'UTR" [p.6 lines 20-21]. It is not clear from the referenced Figure S1A or the methods section how this was calculated.Response: We have added a detailed protocol about the above analysis in the method section.3. As m6A sites themselves are enriched in the CDS and 3'UTR, this has to be taken into account in order to make such a statement. This becomes even more important, as the proportions of variants in the different regions in Fig. S1 do not add up. This suggests that the authors were not restricting their analysis to variants that map to mRNA. This is an issue, as most of the non-m6A-variants seem to be located outside of mRNA transcripts, whereas the m6A-variants naturally occur almost exclusively in mRNAs.Response: The m6A-association variants observed in our analysis were mainly enriched in the CDS and 3' UTR, which is in line with the fact that m6A sites were more likely to occur in CDS and 5'-UTR regions. Our original intention was to illustrate that the identified m6A-association variants agreed well with the known characteristics of m6A sites, indicating the robustness of our predictor. Therefore, we have modified our statement and made this point more clearly. As for Fig. S1, we have reanalyzed the data and restricted the test to variants that map to mRNA. The original Fig. S1 was changed to Fig. S2. To show entire results, the 5'-UTR is added (Table S1). Now proportions in different regions should be summed up to 1.4. Therefore, without proper filtering two very different sets of variants are compared in the downstream analyses. This can be seen in

Figure 3A, where the conservation of m6A-associated variants is analyzed. If most of the non-m6A-variants are located in intergenic regions, it is totally expected that they are less conserved. Therefore, in order to test whether there is indeed a higher selective pressure on the m6A-variants as compared to the non-m6A-variants, they have to be compared to the non-m6A-variants in mRNAs (preferably even in the same exons).Response: In fact, the analyses of m6A-association variants were performed in exonic regions. We have already filtered the intronic and intergenic regions before the analysis in Fig. 3A. To clarify this point more clearly, we added some descriptions in the method section. Furthermore, we also tested the conservative and deleterious differences between m6A-association variants and non-m6A variants in the same exons, the result of which were shown in the modified Fig. 3. Interestingly, the statistical test was still significant even under this strict condition. We have revised our manuscript as shown below.Reviewer's response to 2.), 3.) and 4.):The individual analyses have now been described in more detail. Also, my concern that intergenic variants were included in the analysis of m6A-associated variants was addressed. However, I still think that the claim that "m6A-associated variants [...] could be driven by positive selection" (p. 8 line 17) is not fully supported by the current data.The reason is that there is still significant positional bias in the compared groups (m6A variants vs. non-m6A variants). While I agree that the enrichment of m6A-variants in CDS (and 3'UTR) points towards their validity of being m6A sites, this makes a comparison with the non-m6A variants difficult. The problem is identical to the one arising from comparing variants in genic and intergenic regions: The two regions compared are under different selective pressure. The same holds true also for coding sequence vs untranslated regions, as the latter are typically less conserved. Thus, the enrichment of m6A-variants in the CDS (see the supplementary table 1) leads to an oversampling of this region that predicts that these variants are on average more conserved and more deleterious.Therefore, as long as the oversampling of CDS is not taken into account, it is difficult to conclude that m6A-variants are indeed more conserved than their non-m6A counterparts. One way to control for this might be to analyze CDS and UTRs separately or to subsample the sites so that each set has a similar proportion of these regions.Minor comments:All minor comments except #3 and #5 have been adequatly addressed.3. In the introduction, the link between FTO SNVs and obesity/diabetes [p. 4 lines 9-10] needs to be toned down, as the FTO intronic variants likely act via changing the expression of neighboring genes and not FTO itself (Smemo et al., 2014; 10.1038/nature13138).Response: Thanks for your comments. We have revised this sentence as your suggestion.Reviewer's response:The sentence was now changed to "The mutation on FTO, an m6A demethylase, can change the expression level of neighboring genes and therefore leading to obesity and type 2 diabetes [27]." (p4. line 9). This misses the point that mutations in FTO have not been linked to obesity and type 2 diabetes. The loss of function mutations in the protein coding region that interfere with the demethylase activity of FTO lead to a severe developmental phenotype (as the authors correctly state on p4. lines 11-17). The link of the FTO locus with obesity comes from intronic SNVs, which are reported to regulate expression of neighboring genes rather than FTO activity. Thus, a direct link between FTO activity and obesity/diabetes is still elusive.5. There are a lot of typos throughout the manuscript, even in the figures (Fig. 3B).Response: We are sorry for the typos. We have gone through our manuscript again and corrected all the typos in the main text.Reviewer's response:There are still some typos (e.g. p7 line 58 "To further dissert the functional role" or p. 8 line 33 "have revealed many diseases related variants").

Level of Interest

Please indicate how interesting you found the manuscript: An article of importance in its field

Quality of Written English

Please indicate the quality of language in the manuscript: Needs some language corrections before being published

Declaration of Competing Interests

Please complete a declaration of competing interests, considering the following questions:

- Have you in the past five years received reimbursements, fees, funding, or salary from an organisation that may in any way gain or lose financially from the publication of this manuscript, either now or in the future?
- Do you hold any stocks or shares in an organisation that may in any way gain or lose financially from the publication of this manuscript, either now or in the future?
- Do you hold or are you currently applying for any patents relating to the content of the manuscript?
- Have you received reimbursements, fees, funding, or salary from an organization that holds or has applied for patents relating to the content of the manuscript?
- Do you have any other financial competing interests?
- Do you have any non-financial competing interests in relation to this paper?

If you can answer no to all of the above, write 'I declare that I have no competing interests' below. If your reply is yes to any, please give details below.

I declare that I have no competing interests.

I agree to the open peer review policy of the journal. I understand that my name will be included on my report to the authors and, if the manuscript is accepted for publication, my named report including any attachments I upload will be posted on the website along with the authors' responses. I agree for my report to be made available under an Open Access Creative Commons CC-BY license (http://creativecommons.org/licenses/by/4.0/). I understand that any comments which I do not wish to be included in my named report can be included as confidential comments to the editors, which will not be published.

I agree to the open peer review policy of the journal

To further support our reviewers, we have joined with Publons, where you can gain additional credit to further highlight your hard work (see: https://publons.com/journal/530/gigascience). On publication of this paper, your review will be automatically added to Publons, you can then choose whether or not to claim your Publons credit. I understand this statement.

Yes
